# Chloroplast and nuclear DNA exchanges among *Begonia* sect. *Baryandra* species (Begoniaceae) from Palawan Island, Philippines, and descriptions of five new species

**DOI:** 10.1371/journal.pone.0194877

**Published:** 2018-05-02

**Authors:** Mark Hughes, Ching-I Peng, Che-Wei Lin, Rosario Rivera Rubite, Patrick Blanc, Kuo-Fang Chung

**Affiliations:** 1 Royal Botanic Garden Edinburgh, Edinburgh, United Kingdom; 2 Research Museum and Herbarium (HAST), Biodiversity Research Center, Academia Sinica, Taipei, Taiwan; 3 Herbarium of Taiwan Forestry Research Institute (TAIF), Taiwan Forestry Research Institute, Taipei, Taiwan; 4 Department of Biology, University of the Philippines Manila, Padre Faura, Manila, Philippines; 5 CNRS, 3 rue Michel-Ange, Paris, France; National Cheng Kung University, TAIWAN

## Abstract

The Philippine island of Palawan is highly biodiverse. During fieldwork there in 2011 & 2014 we found five unknown species in the large genus *Begonia*. The species are similar in their rhizomatous stems, four-tepaled flowers, inferior two- or three-locular ovaries with bilamellate placentas, and are assignable to *Begonia* sect. *Baryandra*. Our observations support the recognition of these as five new species endemic to Palawan: *B*. *elnidoensis*, *B*. *gironellae*, *B*. *quinquealata*, *B*. *tabonensis* and *B*. *tenuibracteata* which are described here. The five new species were added to phylogenies based Bayesian analysis of nrDNA (ITS) and chloroplast DNA (*ndhA*, *ndhF-rpl32*, *rpl32-trnL*, *trnC-trnD*), along with 45 other allied ingroup species. A majority of the species show incongruent positions in the two phylogenies, with evidence of prevalent chloroplast capture. Models show chloroplast capture is more likely in plant populations with high levels of inbreeding following a reduction in selfing rate after hybridisation; we suggest that this is a possible explanation for the massive amount of chloroplast exchange seen in our phylogeny, as *Begonia* species often exist as small isolated populations and may be prone to inbreeding depression. Our data also indicate a level of nuclear genetic exchange between species. The high prevalence of hybrid events in *Begonia* is potentially an important factor in driving genomic change and species evolution in this mega-diverse genus.

## Introduction

Palawan is the only island province in the Philippines with more than 50% of its forest cover intact and it has been estimated that the island contains 1,522 species of flowering plants with 15–20% endemism [1]. The island is key not only as a conservation area, but also as a biogeographic nexus of great influence in the evolution of the eastern Malesian biota [2–4]. Recently our knowledge of the herbaceous diversity of the island has been augmented by the discovery of 11 new species of *Begonia* L. [5–8], nearly tripling the original number known to a total of 17 species. Of these, only *Begonia mindorensis* Merr. is distributed in both Luzon and Palawan; all the other 16 species are endemic to Palawan [9]. During recent fieldwork to the municipalities of El Nido and Quezon and Puerto Princesa City on Palawan, the authors found five species of *Begonia* which did not correspond to known taxa. Based on a study of relevant literature, type collections, as well as observation of living plants both from the field and in the greenhouse, we confirm that the five unknown *Begonia* are new species endemic to Palawan, which are here described and illustrated. They belong to *Begonia* sect. *Baryandra* A.DC., a section of the genus which has its centre of diversity in the Philippines [10]. Species in this section are rhizomatous, stemless, monoecious herbs, often lithophytic and associated with riverine habitats and waterfalls. The group arrived in the Philippines in the late Miocene, via long-distance dispersal from western Malesia and a point of entry likely to be in the northwestern region of the archipelago [3]. Palawan, Luzon, and Panay all bear early-branching lineages from this initial colonization [3]. The discovery of further diversity of the species rich genus *Begonia* on Palawan presents an opportunity to investigate the dynamics of the evolution of the highly endemic flora of the island.

*Begonia* is a mega-diverse genus of 1839 species [9], with a very high proportion of microendemics and hotspots of diversity in the Andes and Southeast Asia. This pattern of diversity begs an explantion; it has been suggested that the emergence of new habitats during the uplift of the Andes and the rapid geological evolution of the Malesian region has driven the diversification [11,12]. During exposure to new habitats, *Begonia* may be predisposed to forming new, narrowly endemic species due to remarkably low levels of gene flow, leading to weak selection pressures being able to shape new taxa in the absence of genetic contact with other populations [13]. Diversification may be further driven by as yet poorly understood characteristics of the *Begonia* genome, which may have an underlying genetic instability as evidenced by the highly variable chromosome number in the genus. This is suggestive of hybridisation followed by polyploidisation, with frequent chromosome fission and fusion leading to the observed dysploid variation in chromosome number in *Begonia* [14].

The extent of hybridisation in *Begonia*, at least in some clades, is only starting to become apparent from phylogenetic evidence. A majority of previous phylogenetic studies of *Begonia* have relied on a single genome for their data, usually the chloroplast [8,11,12,15] or nrITS [10,16–19]. Some studies have analysed combined datasets, using nrITS and various chloroplast loci [20–24]. Some of these studies did highlight incongruence between the combined loci, either significant incongruence according to an ILD test [22] or topological but unsupported or moderately supported incongruence [20,24]. Only two studies did not uncover any significant incongruence between nuclear and chloroplast datasets [21,23]; however these both used the *trnL* intron from the chloroplast which tends to give low resolution in *Begonia* phylogenies. Two studies have presented phylogenies from two different genomes which showed considerable amounts of supported phylogentic incongruence in *Begonia*. Goodall-Copestake et al. [25] found incongruence between the chloroplast and mitochondrial phylogenies in a genus-wide study, and Hughes et al. [3] between nrITS and chloroplast phylogenies in a study of *Begonia* sect. *Baryandra*. It is this latter study to which we added further sampling of the new species mentioned above, and the further incongruence we uncovered prompted this manuscript.

Here we investigate the phylogenetic relationships of the five new *Begonia* species from Palawan and their relatives in *Begonia* sect. *Baryandra* using DNA sequence markers from the chloroplast genome and the nuclear genome. The chloroplast genome is maternally inherited in *Begonia* [26], whereas the markers we use from the nuclear genome (nuclear ribosomal DNA internal transcribed spacers (nrDNA ITS)) are diploid and bi-parentally inherited, and are located in the 45S ribosomal RNA genes. The few previous studies which have demonstrated phylogenetic incongruence between various genomic markers in *Begonia* hint that hybridisation has the potential to be important in the generation of the massive diversity of the genus. The markers from different genomes allow us to gain further insight into the prevalence of past hybridisation events in the evolution of the species in *Begonia* sect. *Baryandra*.

## Materials and methods

### Ethics statements

Our field studies in Palawan Island, Philippines, have been permitted by Palawan Council for Sustainable Development, the Puerto Princesa Subterranean River National Park Protected Area Management Board, the Puerto Princesa City Council, the El Nido-Taytay Managed Resource Protected Area Management Board, the El Nido Municipal Council, and the Department of Environment and Natural Resources. Since this species is currently undescribed, it is inevitable not included in the protecting list.

### Morphology

Morphological observations were initially made from wild populations. Further investigations into the morphology of the new and allied taxa were made it the laboratory based on literature, herbarium specimens, and living plants; measurements in the descriptions are based mainly on living plants, supplemented by data from dried specimens. The five new species were studied together with the most closely allied members of the sect. *Baryandra*. *Begonia elnidoensis* was compared to *B*. *wadei* Merr., *B*. *gironellae* to *B*. *cleopatrae* C.Coyle, *B*. *quinquealata* to *B*. *suborbiculata* Merr., and both *B*. *tenuibracteata* and *B*. *tabonensis* were compared to *B*. *mindorensis*. Type specimens (*B*. *elnidoensis Peng 23508*, *B*. *gironellae Peng 24579*, *B*. *quinquealata Peng 24588*, *B*. *tabonensis Peng 24538* and *B*. *tenuibracteata Peng 23452*) are deposited at PNH and HAST.

### Nomenclature

The electronic version of this article in Portable Document Format (PDF) in a work with an ISSN or ISBN will represent a published work according to the International Code of Nomenclature for algae, fungi, and plants [27], and hence the new names contained in the electronic publication of a PLoS article are effectively published under that *Code* from the electronic edition alone, so there is no longer any need to provide printed copies.

In addition, new names contained in this work have been submitted to International Plant Name Index (IPNI), from where they will be made available to the Global Names Index. The IPNI Life Science Identifiers (LSIDs) can be resolved and the associated information viewed through any standard web browser by appending the LSID contained in this publication to the prefix http://ipni.org/. The online version of this work is archived and available from the following digital repositories: PubMed Central, LOCKSS, and The *Begonia* Resource Centre (http://padme.rbge.org.uk/begonia/).

### Molecular phylogenetics

Molecular phylogenies were constructed based DNA sequences from the chloroplast genome (four noncoding regions *ndhA* intron, *ndhF-rpl32* spacer, *rpl32-trnL* spacer, *trnC-trnD* spacer) and the nuclear genome (nuclear ribosomal internal transcribed spacers ITS1 and 2 with the 5.8S gene). Taxon sampling was based on that of Hughes et al. [3], supplemented with 14 new accessions including all the new species described herein. The ingroup, *Begonia* sect. *Baryandra*, has been found to be strongly supported as monophyletic [3,10]; the outgroup sampling for both phylogenies matches that of Hughes et al. [3]. A total of 91 accessions were sampled for the chloroplast regions, representing 67 taxa including outgroups, and 69 accessions representing 50 taxa (including outgroups) for the nuclear region. Voucher and GenBank accession information is listed in S1 Table. The DNA extraction, PCR and sequencing were carried out as in Hughes et al. [3]. The following bases were excluded from the chloroplast alignment because of missing data at the ends of regions, or alignment uncertainty due to long mononucleotide repeats: 1–10, 1390–1540, 2510–3050, 4530–4645, 4746–4910, 4943–4997, 5720–5810, 6970–7125, 8050–8089; and from the nuclear alignment bases 494–548 were excluded. Appropriate models of DNA sequence evolution for the aligned datasets were assessed with the program jModeltest 2.1.3 [28] using the corrected Akaike information criterion (AICc) to estimate the model with the closest fit to the data. For both the chloroplast and nuclear alignment, the GRT+G+I model was the most probable (AIC weight 1.00 and 0.66 respectively).

Bayesian phylogenetic analyses were carried out separately on the chloroplast and nuclear data sets using the program MrBayes 3.2.6 [29]. Each data set was treated as a single partition, analysed under the appropriate model of sequence evolution and the default parameters of two runs with four chains each, run for 10 000 000 generations with a sample tree taken every 10 000 generations. The convergence of the MCMC chains of the two runs was assessed by inspection of the trace plots of parameters using Tracer ver. 1.6 [30]. The effective sample sizes (ESS) of all parameters were > 200, indicating that each parameter was sampled satisfactorily. The first 25% of sampled trees were discarded as burn-in, and the remainder summarized as a maximum clade credibility tree. Trees were visualised using the APE package [31]. Strict and semi-strict consensus trees combining the two analyses were constructed in PAUP [32].

### Incongruence tests

Alignments with matching sampling for both chloroplast and nuclear markers were constructed for use in conducting an Incongruence Length Difference test (ILD) [33] and measures of internode certainty (IC) [34]. The ILD test was conducted using PAUP, using 100 replicates based on heuristic searches with TBR branch swapping, each with 10 random addition replicates and Maxtrees set to 100. In order to further examine topological incongruence between the datasets, post burn-in samples of trees resulting from the Bayesian phylogenetic analysis of the chloroplast and ITS data were combined into a single majority rule consensus (MRC) tree with IC values for each node using RaxML [35]. The IC metric (scaled between 0 and 1) represents the certainty for each internal branch, taking into account the frequency of the most prevalent conflicting bipartition in the population of trees used to make the consensus. For example, if there are two conflicting bipartitions between the chloroplast and ITS derived topologies, each present at 100% frequency in the different datasets, then IC = 0, reflecting the complete incongruence between the genomes. A bipartition with 100% frequency in trees derived from the chloroplast dataset and 50% frequency in the ITS dataset, contradicted by 50% of the remaining trees in the ITS dataset would have an IC = 0.18. Comparing all post burn-in trees allows us to further investigate the full range of topologies produced by the two separate analyses, rather than just comparing a separate consensus of each.

## Results

### Molecular phylogenetics

**Fig 1:**
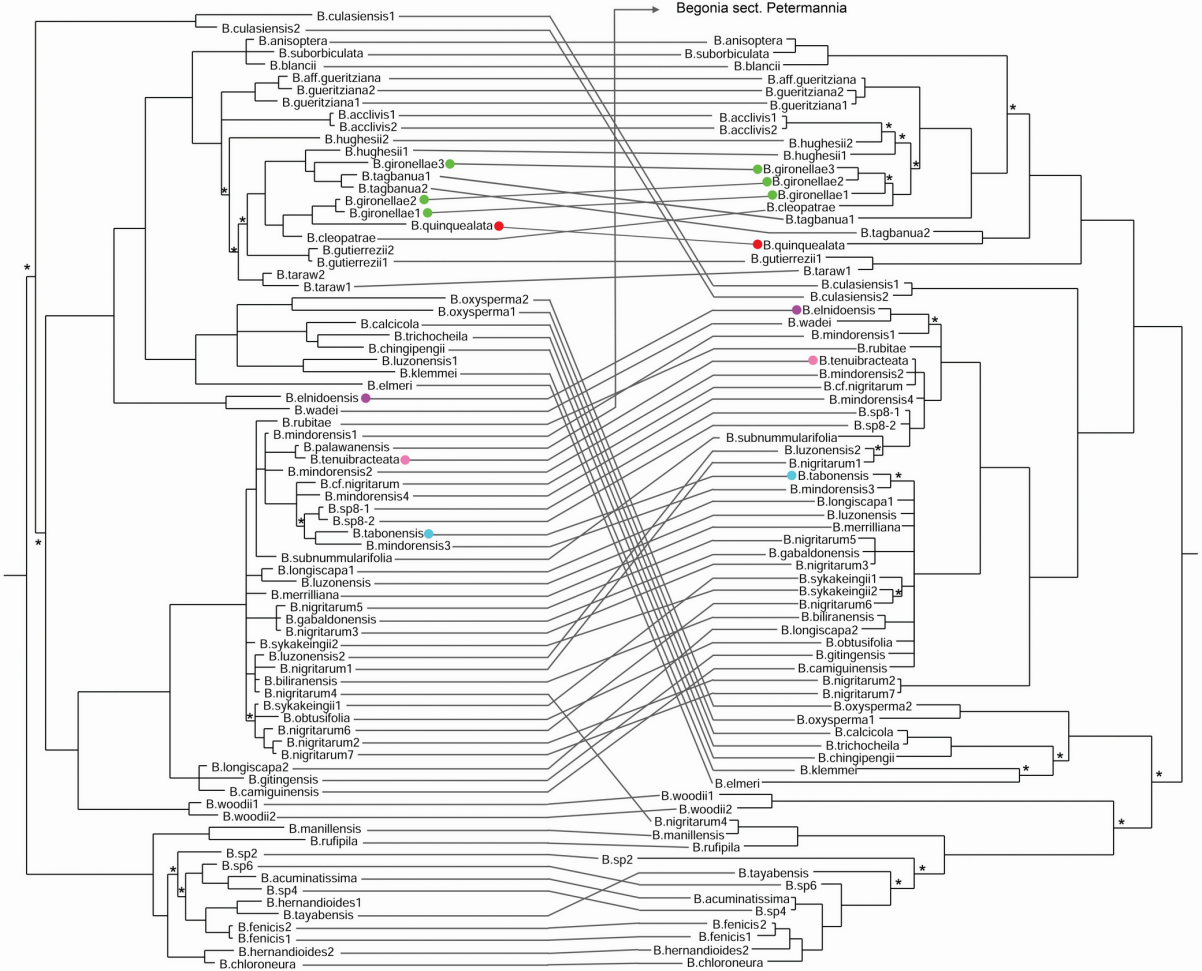
Tanglegram comparing nuclear and plastid phylogenies of *Begonia* sect. *Baryandra*. Based on Bayesian analyses of chloroplast data (left) and nrDNA ITS data (right); trees are 50% majority rule consensus trees; asterisks mark clades with PP< 0.95; outgroups not shown; scale bars show number of substations per site in the alignment. The species described as new are highlighted by coloured circles.

The ITS alignment consisted of 853 bp in length, with 299 of these being parsimony-informative. The chloroplast alignment consisted of 6764 bp in length, with 370 of these being parsimony informative. A majority of the species sampled in our *Begonia* sect. *Baryandra* phylogenies show some incongruence between their placements in the chloroplast and nuclear phylogenies (Fig 1 and S1 Fig). No readable ITS sequence could be obtained for the terminal *B*. *hernandioides*1, as the electropherograms showed polymorphisms at most sites. The ITS electropherograms for terminals *B*. *nigritarum*3, *B*. *tabonensis*, *B*. *wadei*, *B*. *woodii*1 and *B*. *woodii*2 showed some polymorphism towards the end of the reads, however good sequence data was obtained for all the alignment for these taxa with the exception of portions of the 5.8S gene and the ITS2 spacer.

### Incongruence tests

The ILD test, as expected, showed significant incongruence between the chloroplast and ITS datasets (P = 0.01). Only two clades of more than three taxa were present in the MRC tree combining trees from both datasets, one consisting of species mainly from the northern Philippines (B. fenicis1 to B. sp2, S1 Fig) and one of species endemic to Palawan (B. taraw1 to B. hughesii1, S1 Fig). Within the latter clade (i.e., Palawan clade), the following clades were resolved in the MRC tree: *B*. *gueritziana* and *B*. aff. *gueritziana* (IC = 1.00); *B*. *anisoptera*, *B*. *blancii* and *B*. *suborbiculata* (IC = 1.00); *B*. *cleopatrae* and *B*. gironellae (IC = 0.00); *B*. *acclivis* and *B*. *hughesii* (IC = 0.18); *B*. *taraw* and *B*. *gutierrezii* (IC = 0.00). Within the northern Philippine clade, three clades were resolved: *B*. *acuminatissima* and *B*. *sp*. 4 (IC = 1.00); *B*. *chloroneura* and *B*. *hernandioides* (IC = 0.00); *B*. *fenicis*1 & 2 (IC = 1.00). Within the remainder of the MRC tree, the other resolved pairs of terminals are largely duplicates of the same species, with high congruence (IC = 1.00; *B*. *culasiensis*, *B*. *nigritarum*, *B*. *oxysperma*, *B*. *sp*. 8, *B*. *woodii*).

Given the amount of incongruence between the phylogenies, assessing which species have a similar position in the two analyses is difficult. A large number of species show hard incongruence between our analyses, being in conflicting positions with support of PP >0.95. We split these into two categories, depending on the spread of the differing positions in the two analyses. Firstly, there are species where the incongruence could potentially be due to lineage sorting, as the branch lengths are relatively short and only one node is involved. These are: *B*. *calcicola*, *B*. *chingipengii*, *B*. *chloroneura*, *B*. *fenicis*, *B*. *hernandioides*, *B*. *submummularifolia*, *B*. *tabonensis*, *B*. *trichocheila*, *B*. *sp*. 4, *B*. *sp*. 6. Secondly, there are species in which the incongruent phylogenetic positions are more likely to be due to genomic exchange via hybridisation, as there are two or more nodes different in the positions of the species. These are: *B*. *anisoptera*, *B*. *biliranensis*, *B*. *blancii*, *B*. *camiguinensis*, *B*. *elnidoensis*, *B*. *gironellae*, *B*. *gitingensis*, *B*. *gutierrezii*, *B*. *hughesii*, *B*. *longiscapa*, *B*. *luzonensis*, *B*. *mindorensis*, *B*. *nigritarum*, *B*. *palawanensis*, *B*. *quinquealata*, *B*. *suborbiculata*, *B*. *tagbanua*, *B*. *taraw*, *B*. *tayabensis*, *B*. *wadei*, *B*. *sp*. 8, *B*. *acuminatissima*. Some taxa (*B*. *anisoptera*, *B*. *blancii*, *B*. *suborbiculata*) could potentially be affected by both hybridisation early in their history, leading to marked incongruence, whilst also presenting less marked and highly nested topological incongruence within their sub-clade, potentially the result of lineage sorting.

## Discussion

The five new species described here show that Palawan is biodiverse and underexplored; four of the species are from nearby localities in the El Nido municipality, and so further exploration in other parts of the island is likely to reveal additional novelties. The ongoing discovery of biodiversity on Palawan highlights the need to preserve its current forest cover and ensure protected areas are sustainably managed. The declaration by the Palawan Council for Sustainable Development (PCSD) of the Cleopatra’s Needle Critical Habitat [36] status is particularly welcome. This is the largest area of critical habitat designation in the Philippines and a landmark for conservation in the region.

The evolution of the *Begonia* diversity on Palawan is a consequence of relict lineages from the late Miocene combined with recent Pliocene-Pleistocene diversification [3]. The increase in sampling in this study has shed further light on the phylogenetic relationships of Palawan *Begonia*, and has highlighted remarkable levels of incongruence between nuclear and plastid derived phylogenies. Incongruence between plastid and nuclear phylogenies can be attributed to either incomplete lineage sorting or chloroplast capture [37]. Incomplete lineage sorting is the inheritance of alleles during the speciation process whose genealogy differs from the bulk of the genome which follows the species phylogeny. It is a phenomenon which occurs between recently or previously rapidly diverged species. Chloroplast capture occurs when the chloroplast genome is introgressed from another species, leading to a genealogical mismatch to the nuclear genome. Markers from the nuclear genome would give the correct species tree, whereas chloroplast markers would reflect the phylogenetic position of the plastid donor. Chloroplast capture can happen between either recently diverged or more distantly related species [38].

**Fig 2:**
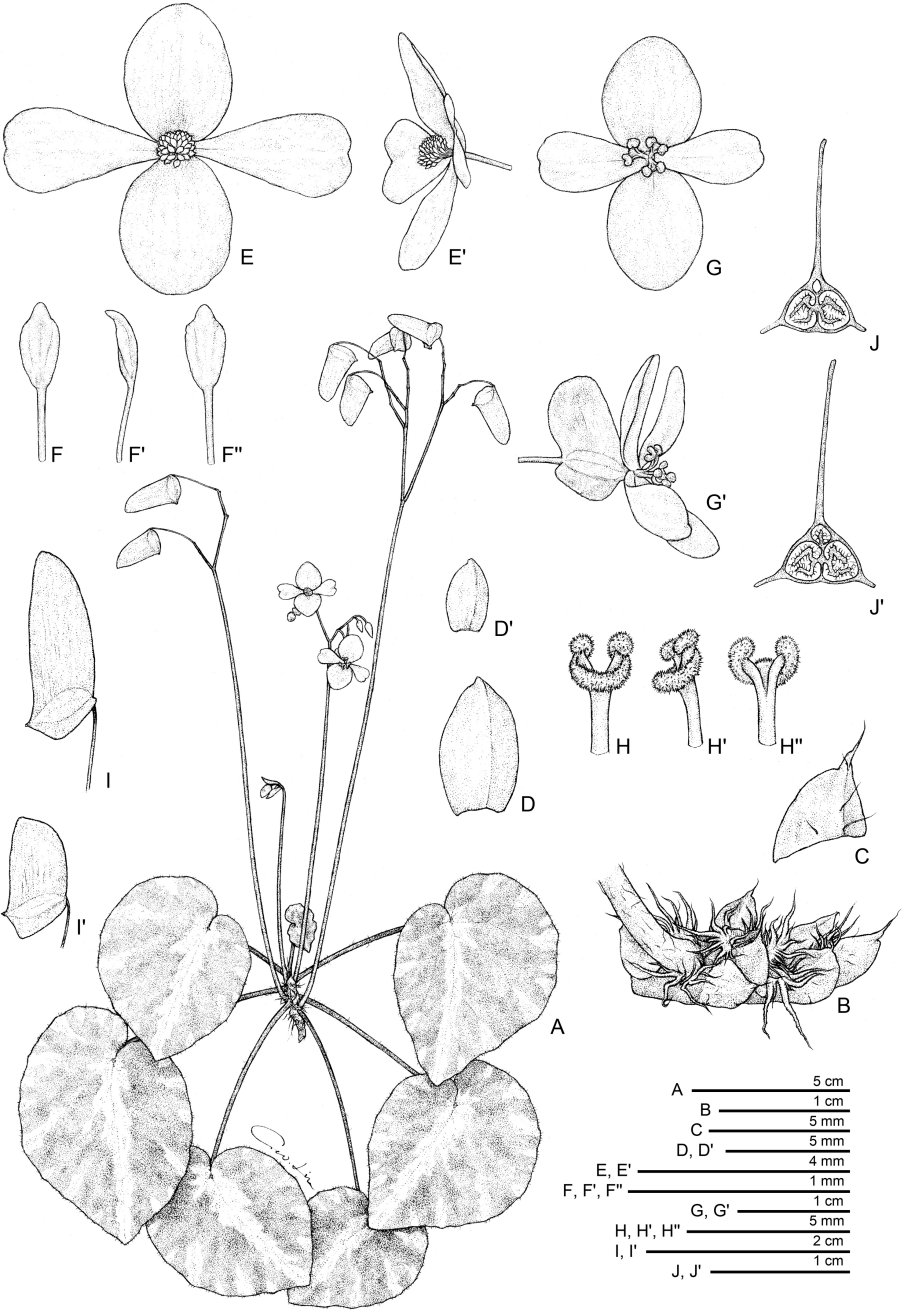
*Begonia gironellae* C.I Peng, Rubite & C.W.Lin. **A,** habit; **B,** rhizome, showing stipules and fleshy hairs at petiole base; **C,** stipule; **D, D',** bracts at lowermost and uppermost parts of inflorescence; **E, E',** staminate flower, face and side views; **F, F', F'',** stamen, dorsal, side and ventral views; **G, G',** pistillate flower, face and side views; **H, H', H'',** style and stigmatic band, side, dorsal and ventral views; **I, I',** capsules; **J, J',** serial cross section of an ovary. All from *Peng 24579*.

**Fig 3:**
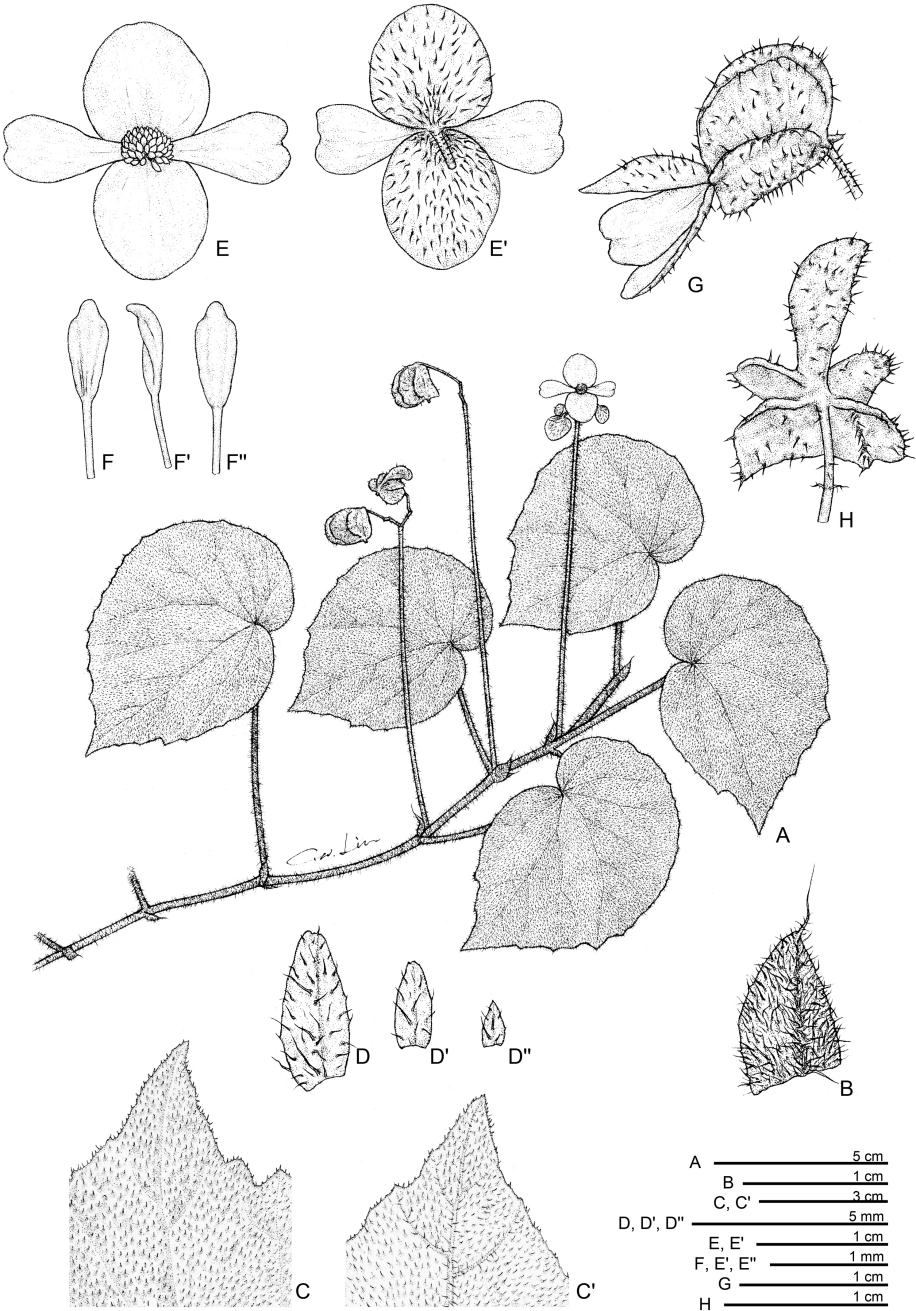
*Begonia quinquealata* C.I Peng, Rubite & C.W.Lin. **A,** habit; **B,** stipule; **C, C',** portion of leaf, upper and lower surface; **D, D', D'',** bracts at lowermost to uppermost parts of inflorescence; **E, E',** staminate flower, face and back views; **F, F', F'',** stamen, ventral, side and dorsal views; **G,** immature pistillate flower; **H,** capsule, bottom view, showing 5-wings. All from *Peng 24588*.

We estimate that approximately half of the 49 species in our phylogeny show phylogenetic incongruence which is likely to be due to hybridisation rather than stochastic lineage sorting, owing to the deep incongruence shown in the different gene trees. Chloroplast capture is expected to be more commonly observed than nuclear gene exchange in plants, due to the smaller effective population size of the haploid plastid genome, making it more likely that a foreign chloroplast genotype will become fixed in populations of the recipient species [39]. Our phylogeny demonstrates that in *Begonia* sect. *Baryandra* prevalent chloroplast capture seems to be the case; for example, the three samples of *B*. *gironellae* are monophyletic in the nrDNA phylogeny, whilst appearing in two positions in the chloroplast phylogeny (Fig 1); the plastid genome of *B*. *gironellae*3 is likely to be captured from a different lineage. Also, *B*. *quinquealata* appears in a clade of species with straight fruit wings (e.g., Fig 2I) in the chloroplast phylogeny, whereas in the rDNA phylogeny it sits morphologically comfortably in a grade with several species sharing markedly cucullate fruit wings (e.g., Fig 3H), and hence the rDNA represents the most probable species tree. *Begonia palawanensis* and *B*. *tenuibracteata* share a very similar chloroplast haplotype, yet they belong to different sections of the genus (sects. *Petermannia* and *Baryandra* respectively). The ITS sequence data from *B*. *palawanensis* places it outside the outgroups used for the analysis, and in a clade reflecting its morphology and taxonomy, with other members of *Begonia* sect. *Petermannia* from Borneo, the Philippines and New guinea (analysis not included here). The samples for these two species were taken from nearby localities near Salakot Falls, and it is probable that the population of *B*. *palawanensis* has captured the chloroplast from adjacent populations of *B*. *tenuibracteata*. Hence it seems that a large proportion of the 49 species we sampled from Palawan have acquired a foreign plastid at some point during their evolution. Chloroplast capture may be aided in *Begonia* by the very weak barriers to hybridisation in the genus, with F_1_ hybrids being readily made between even distantly related species [14]. However, natural F_1_ hybrids in *Begonia* have frequently been found to be pollen sterile [40, 41] and even crosses between isolated populations of the Mexican species *B*. *heracleifolia* have been found to have reduced pollen fertility [42]. Despite these observed barriers to hybrid fertility, in order to explain the prevalence of chloroplast capture it seems likely that in many cases it infers a fitness benefit. *Begonia* species exist in isolated populations connected by only low levels of gene flow [13, 43, 44], potentially leading to inbreeding depression. A model of chloroplast capture by Tsitrone et al. [38] demonstrates that capture is facilitated when the selfing rate is reduced in hybrids (for example by reduced pollen fertility) in populations with strong inbreeding depression. This would lead to increased introgression from pollen with the ‘native’ genotype, with potentially a relative increase in female fitness in individuals with a foreign chloroplast genotype. Most *Begonia* species are fully self-compatible and partially selfing [13, 42]; if inbreeding depression is significant in the genus, this could explain the high degree of chloroplast capture we have observed in Palawan *Begonia*. The very high levels of population isolation observed in *Begonia* also mean that chloroplast capture is more likely from a neutral perspective than in more panmictic groups, due to the reduced flux of genes from other populations which could ‘flush out’ foreign haplotypes [45]. Hybridisation seems to be linked to geographic proximity, as in the cases of *B*. *palawanensis* and *B*. *tenuibracteata* described above, of *B*. *elnidoensis* and *B*. *mindorensis* which have overlapping distributions in northern Palawan, and of the within-Palawan hybridisation evidenced by the other new species described here. This scenario is congruent with the geographically limited gene flow recorded for *Begonia* species [13]. There is no evidence for morphological intermediacy in the species observed to have undergone past hybridisation; for example *B*. *elnidoensis* is morphologically and ecologically very divergent from *B*. *mindorensis*, and the three individuals of *B*. *gironellae* with differing phylogenetic histories are not morphologically dissimilar. We found no evidence for morphologically intermediate F1 hybrids in the field.

Some of the incongruence observed is likely to be due to nrDNA capture, as there are some species in which the chloroplast phylogeny reflects the most probable species tree in terms of morphology and distribution, with the nrDNA marker showing an incongruent placement. This is the case in *Begonia wadei* and *B*. *elnidoensis*, which are succulent stemmed species ecologically aberrant for the genus. Their placement at the base of a Palawan endemic clade which began diverging in the late Miocene [3] is much more plausible than their highly nested placement in a clade of rhizomatous forest-dwelling species predominantly from Luzon and Panay. The two divergent nrDNA genotypes possessed by *B*. *tagbanua* are very divergent; although potentially they could represent ancestral diversity, it is more plausible that the nrDNA genotype of *B*. *tagbanua*2 was captured from a different lineage.

The nrDNA genes are arranged in tandem repeats on regions of chromosomes known as nucleolus organiser regions (NOR). Nucleoli form within the nucleus around the NOR’s, and are responsible for ribosome production. The number of major NOR loci in plants has been found to vary from 1 to 32, although a majority have one or two [46]. DNA sequences of the ribosomal genes and their associated spacers are generally treated as a single locus in the context of phylogenetic analysis, due to concerted evolution homogenising the sequences within and between loci [47]. If we assume *Begonia* is typical amongst angiosperms in having one or two NOR loci, it means that we have detected nuclear genome exchange in at least two species on Palawan using a marker on just one or two chromosomes. Hence it would seem either (i) the NOR loci are preferentially inherited or selected for during hybridisation events, or (ii) hybridisation has been rampant among Palawan *Begonia*; the latter would seem more likely. The forces driving the large amount of chloroplast exchange between *Begonia* species on Palawan seems also to have driven nuclear gene exchange.

Our sampling of a single sequence to represent the likely one or two NOR loci present in *Begonia* is a tiny snapshot of the nuclear genome. If this miniscule sampling is able to identify a signal of nuclear genome exchange between species, it is highly likely to be much more prevalent than we detect here. Previous studies have emphasised how allopatry and absence of gene flow have been important drives of diversification in *Begonia* [13, 22, 44]; however, it seems that ongoing genetic exchange between species is more prevalent than suspected, and begs further investigation. Next generation phylogenomic approaches are needed to reveal how much of a patchwork the nuclear genome of *Begonia* species is, and to identify the scale of between-species genetic exchange from the level of the single gene to entire chromosomes. This may reveal insights into why *Begonia* is so adept at generating new species.

Given the phylogenetic chaos we have uncovered in *Begonia* sect. *Baryandra*, it may seem difficult to pin down a species concept, and certainly a monophyletic species concept is out of the question. However, our taxonomists species concept continues to serve us and other *Begonia* taxonomists well; in the last 2 years 147 new species have been described [9]. Even in the light of molecular phylogenetic data, it seems that careful observation of plants in the field and herbarium remain our most faithful tools for describing the basic units for conservation and research in *Begonia*.

### Taxonomic treatment

#### Key to *Begonia* sect. *Baryandra* on Palawan

As many *Begonia* species are narrow-range endemics, we have added distribution information to help confirm determinations and also highlight new records for other areas (Table 1). Types and representative specimens for most species are available online [9].

**Table 1:** Key to *Begonia* sect. *Baryandra* on Palawan.

**1** Ovaries and fruits conspicuously 5-winged	**2**
**1** Ovaries and fruits 3-winged	**3**
**2** Leaves suborbicular, coriaceous, upper surface glabrous or subglabrous, apex rounded. Northern Palawan, Taytay, 0–50 metres	*B*.* suborbiculata*
**2** Leaves broadly ovate, thickly chartaceous, upper surface densely hirsute, apex acuminate. Puerto Princesa, Salakot Falls, ca. 300 metres	*B*.* quinquealata*, sp. nov.
**3** Leaves variegated	**4**
**3** Leaves not variegated	**11**
**4** Lamina sparsely hairy above	**5**
**4** Lamina glabrous above	**6**
**5** Leaves ovate, broadly shallowly dentate. Southern Palawan, Mt. Mantalingajan & Brookes Point, 130–800 metres	*B*.* acclivis*
**5** Leaves suborbicular, denticulate. Puerto Princesa Underground River NP, 10–200 metres	*B*.* tagbanua*
**6** Leaves spathulate, broadest towards the apex. Northern Palawan, Bulalakaw Falls, 300–400 metres	*B*.* blancii*
**6** Leaves ovate, broadest in the middle	**7**
**7** Abaxial wings on the fruit subequal to lateral wings. Northern Palawan, Busuanga Island, ca. 100 metres	*B*.* rubiteae*
**7** Abaxial wings on the fruit protruded, much wider than lateral wings	**8**
**8** Rhizomes shorter than 5 cm, internodes congested. Puerto Princesa, Tanabag River, ca. 30 metres	*B*. *gironellae*, sp. nov.
**8** Rhizomes to 40 cm long, internodes *ca*. 1 cm apart and evenly spaced. Northern Palawan, Cleopatra’s Needle, ca. 400 metres	*B*.* cleopatrae*
**9** Stem erect, thick-trunked and woody	**10**
**9** Stem rhizomatous or creeping, herbaceous	**11**
**10** Leaves obliquely ovate, main veins *ca*. 13, sparsley hairy beneath. Northern Palawan, coastal Coron Island	*B*.* wadei*
**10** Leaves broadly ovate to subtriangular, main veins ca. 10, glabrous. Northern Palawan, coastal El Nido, Lagen and Miniloc Islands	*B*.* elnidoensis*, sp. nov.
**11** Lamina peltate	**12**
**11** Lamina basifixed	**13**
**12** Petioles with erect 4 mm hairs, internodes ca. 4cm long. Southern Palawan, Lipuun Point and Tawa-Tawa, ca. 30 metres	*B*.* gutierrezii*
**12** Petioles with appressed lanate hairs, internodes 5–10 mm. Puerto Princesa Underground River NP, 5–100 metres	*B*.* taraw*
**13** Lamina very translucent when dry, symmetric to subsymmetric, plants with a small tuber	**14**
**13** Lamina not translucent when dry, distinctly asymmetric, plants rhizomatous	**15**
**14** Leaves usually solitary, less than 6 cm long. Northern Palawan, Malampaya Bay and Lake Manguao	*B*.* woodii*
**14** Leaves several, more than 6 cm long. Northern Palawan, Coron & Culion Islands	*B*.* coronensis*
**15** Fruit 2-locular, one wing distinctly enlarged	**16**
**15** Fruit 3-locular, wings equal or subequal	**18**
**16** Leaves broadly and shallowly dentate to sinuate, with scattered long hairs above. Southern Palawan, Mt. Mantalingajan & Brookes Point, 30–800 metres	*B*.* acclivis*
**16** Leaves minutely denticulate or entire, glabrous or subglabrous above	**17**
**17** Leaves 6–7.5 × 4–5 cm, acuminate, chartaceous. Northern Palawan, Cleopatra’s Needle, ca. 1000 metres	*B*.* wilkiei*
**17** Leaves 7–10 × 5–7.5 cm, acute to shortly acuminate, coriaceous. Puerto Princesa Underground River NP, 5–20 metres	*B*.* hughesii*
**18** Bracts caducous. Southern Palawan, Lipuun Point, Tabon cave area, ca. 30 metres	*B*.* tabonensis*, sp. nov.
**18** Bracts persistent	**19**
**19** Bracts widely to depressed ovate, coriaceous, stamens *ca*. 70. Widespread in Palawan and Mindoro, 300–1300 metres	*B*.* mindorensis*
**19** Bracts ovate to lanceolate, nearly membranaceous, stamens 40–50. Puerto Princesa City, Salakot Falls area, ca. 180 metres	*B*.* tenuibracteata*, sp. nov.

#### Species descriptions

**Fig 4:**
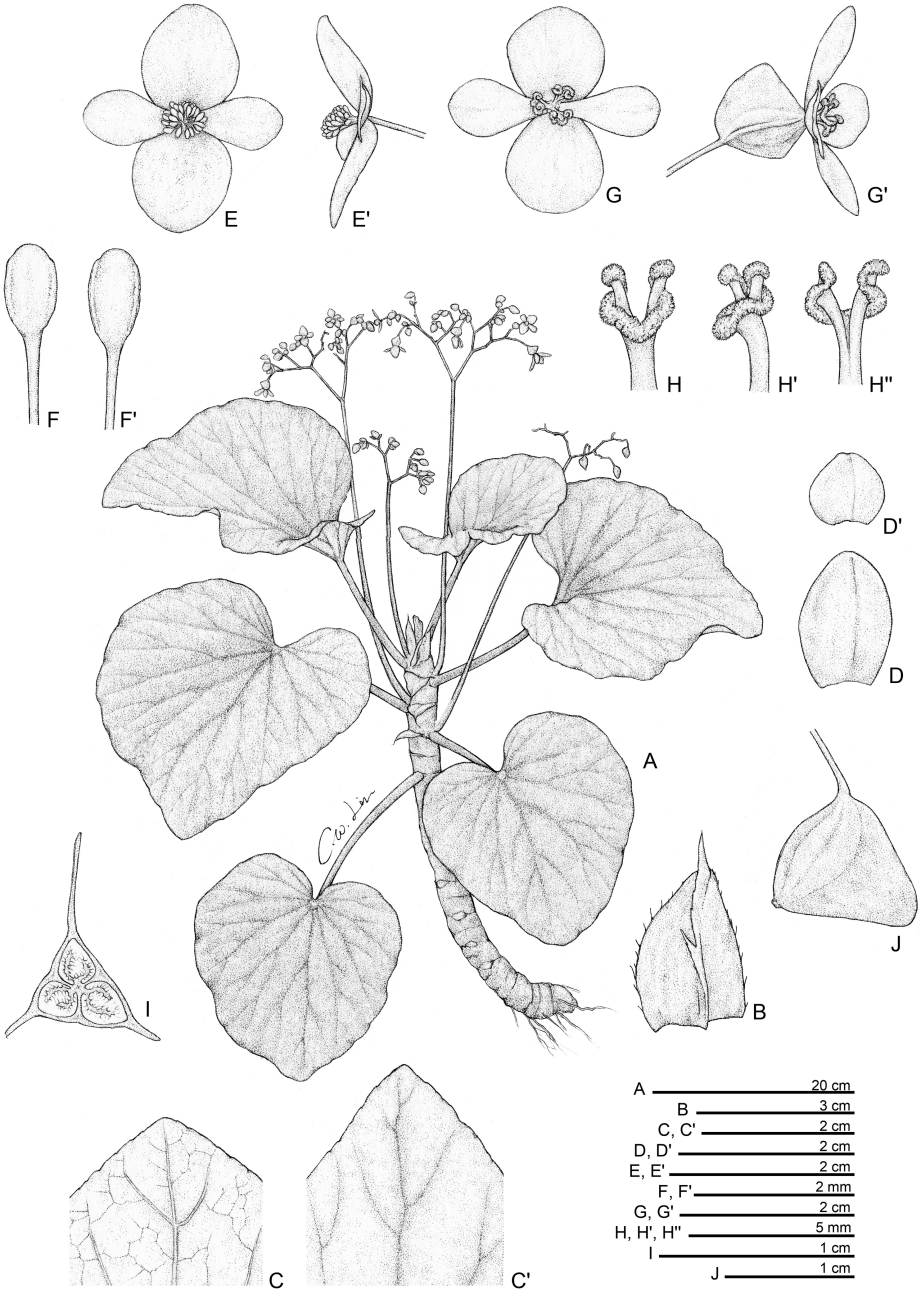
*Begonia elnidoensis* C.I Peng, Rubite & C.W.Lin. **A,** habit; **B,** stipule; **C, C',** portion of leaf, upper and lower surface; **D, D',** bracts at lowermost and uppermost parts of inflorescence; **E, E',** staminate flower, face and side views; **F, F',** stamen, dorsal and ventral views; **G, G',** pistillate flower, face and side views; **H, H', H'',** style and stigmatic band, dorsal, side and ventral views; **I,** cross section of an immature capsule; **J,** capsule. All from *Peng 23508*.

**Fig 5:**
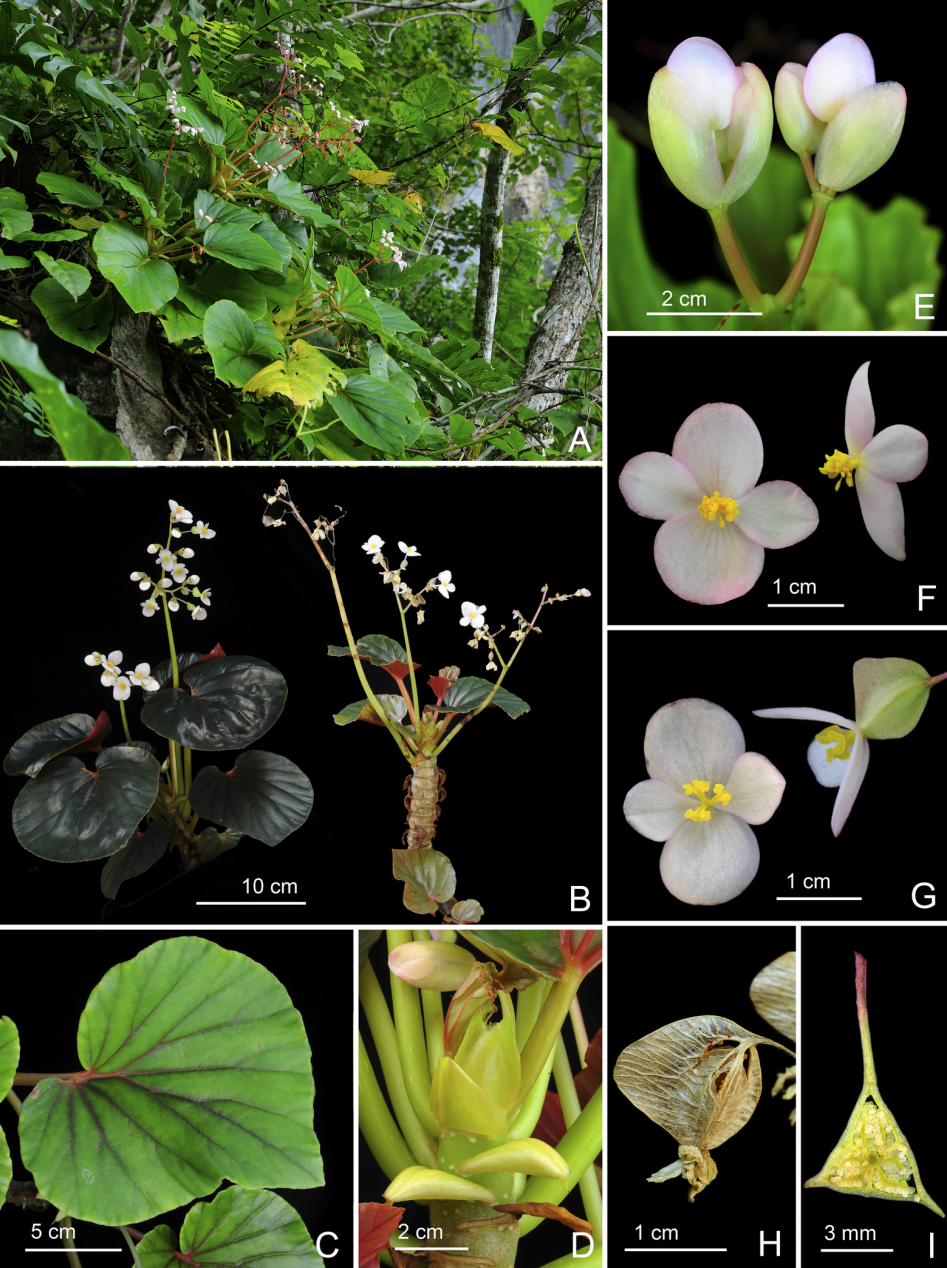
*Begonia elnidoensis* C.I Peng, Rubite & C.W.Lin. **A,** habitat; **B,** habit; **C,** upper surface of leaf; **D,** stem and stipules; **E,** bracts on a developing inflorescence; **F,** staminate flower, face and side views; **G,** pistillate flower, face and side views; **H,** capsule; **I,** cross section of an immature capsule. All from *Peng 23508*.

**Fig 6:**
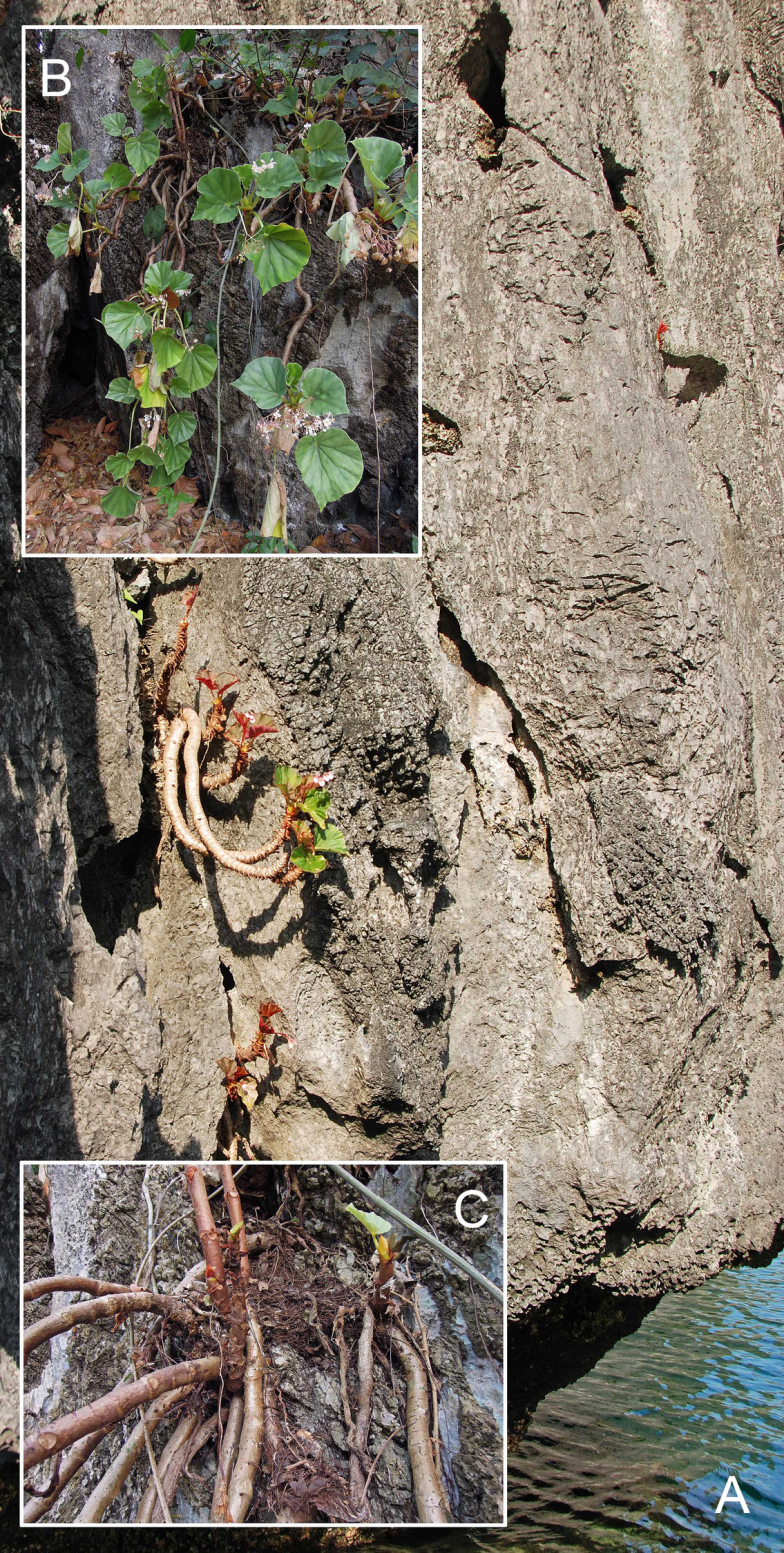
*Begonia elnidoensis* C.I Peng, Rubite & C.W.Lin. **A,** sea-cliff habitat; **B,** habit showing elongated mature stems; **C,** base of the plant showing numerous basally branching stems.

*Begonia elnidoensis* C.I Peng, Rubite & C.W.Lin, sp. nov. [urn:lsid:ipni.org:names:77176485–1] (Figs 4, 5 and 6). Type:—PHILIPPINES. Palawan Island, Poblacion El Nido, on limestone rock face, semiexposed, locally frequent, elevation ca. 2 m, N 11°10'39", E 119°23'28", 4 Nov 2011, *C*.*-I Peng 23508* with K.-F. Chung, C.-I Huang, and R. R. Rubite (HOLOTYPE: PNH; ISOTYPE: HAST).

Monoecious, thick-stemmed herb. Stem unbranched, ascending or pendent from limestone rock face, to 50 cm or longer, to 3 cm thick, internodes 0.5–3 cm long. Stipules persistent, pale yellowish-green or pinkish, widely ovate to ovate-triangular, 1.8–2.6 cm long, 1.2–2.5 cm wide, herbaceous, strongly keeled, glabrous, very sparsely denticulate, velutinous on margin and abaxial midrib, apex shortly aristate, arista *ca*. 2 mm long. Leaves alternate, in a spiral at the top of the stem, petiole terete, pale yellowish-green to reddish, (6.5−)10−25 cm long, (0.5−)0.7−1.5 cm thick, glabrous; leaf blade asymmetric, oblique, widely to very widely ovate, subtriangular,12−30 cm long, 10.5–25 cm wide, broad side 6–17 cm wide, basal lobes cordate, 2–6 cm long, apex obtuse to acute, margin denticulate or subentire; leaf subcoriaceous, succulent, adaxially bright green or dark maroon, veins reddish toward base; abaxially pale green or magenta, veins prominent, red; venation basally *ca*. 8, palmate, midrib distinct, with 3 or 4 secondary veins on each side, other primary veins branching dichotomously or nearly so, tertiary veins reticulate. Inflorescences a axillary, bisexual, cymosely branching panicle 20–40 cm long, peduncle 13–33 cm long, several dichasial cymes arising directly from upper axils of stem, branched 5–6 times, erect or ascending, yellowish-green to pinkish, glabrous; protandrous. Bracts pale yellowish-green, hyaline, deciduous, those at basal node of inflorescence ovate to widely ovate, boat-shaped, 17–30 mm long, 14–20 mm wide, apex obtuse, margin entire; bracts at summit of inflorescence widely ovate, *ca*. 5 mm across, apex attenuate to retuse, margin entire. Staminate flower: pedicel 1.2–2 cm long, glabrous, tepals 4, white to pinkish, glabrous, outer 2 ovate to suborbicular, 1–1.5 cm long, 0.8–1.5 cm wide, inner 2 obovate or oblanceolate, 0.7–1.2 cm long, 0.5–1 cm wide, apex rounded; androecium sub-zygomorphic, *ca*. 0.5 cm across; stamens yellow, 33–45; filaments shortly fused at base; anthers obovate, *ca*. 1.2 mm long, 2-locular, apex apiculate, more or less equal at filaments. Pistillate flower: pedicel *ca*. 2 cm long, glabrous; ovary pale green to pinkish, body trigonous-ellipsoid, *ca*. 8 mm long, 5 mm thick (wings excluded), glabrous; 3-winged, wings unequal, *ca*. 1 cm long, lateral wings narrower, narrowly crescent-shaped to triangular, 0.8–1.5 mm wide, abaxial wing triangular, more or less truncate distally, cuneate proximally, 4.5–7 mm wide, margin entire; ovary 3-locular, bilamellate; tepals 4, white to pinkish, glabrous, outer 2 widely ovate to obovate or suborbicular, 0.9–1.4 cm long, 1–1.2 cm wide, inner 2 widely obovate, 1–1.4 cm long, 0.7–0.9 cm wide, apex rounded; styles 3, short fused at base, yellow, *ca*. 0.5 cm long, stigma spirally twisted. Capsule pendent, pedicel *ca*. 2 cm long, tepals usually deciduous; body trigonous-ellipsoid, *ca*. 1 cm long, 0.7 cm thick (wings excluded), greenish or reddish when fresh; wings unequal, lateral wings 1.5–2 mm wide, abaxial wing to 8 mm wide.

**Distribution and ecology.** *Begonia elnidoensis* is endemic to coralline limestone cliffs in northern Palawan at about sea level (Fig 6). The type specimen was collected from El Nido, and it also occurs in Lagen and Miniloc Islands and other islands and islets adjacent to El Nido as observed by some of the authors during an expedition in 2011. The previously recorded occurrence of the allied species *Begonia wadei* on Miniloc Island [6] is a misidentification, as the collection this is based on (Madulid et al. 27564 [BRIT, L]) is *B*. *elnidoensis*.

After observation of many individuals in different locations, it is clear that this species is potentially immortal through continuous basal emergence of new stems. Considering the leaf scars and internode length, there are alternate sequences of short and long internodes, more pronounced on individuals growing in partly shaded situations, due to the strong tuberisation of the stem in individuals growing on fully exposed sea cliffs making the differences less obvious. About 10 to 12 leaves are produced between long (1.5 to 2 cm long) internodes, and about the same between short internodes (0.5 to 1 cm long). This corresponds to faster and slower growth in the wet and dry season on Palawan. Stem elongation appears to be 20 to 30 cm annually, the longest stems reaching about 120 cm, which are probably four to six years old and likely represent the maximum age. New stems emerge from one of the first short internodes at the base of living older stems and they produce adventitious roots for fixation and nutrition.

Flowering seems to be continuous all the year round, as observed from the couple of scars at each internode; one scar for the petiole and just above one scar for the inflorescence peduncle. Thus this species flowers both in rainy and dry seasons. During fruit maturation, the main inflorescence axis remains turgescent and alive, whereas the secondary axes dry out but remain attached. This junction permits the dry, dehisced capsules to freely shake in the wind. The authors have observed active anemochory in this species.

**Provisional conservation assessment.** This species does not depend on a forest habitat, as it grows crevices in coralline sea cliffs. This habitat is already exposed and hence not under threat from forest clearance, and is also relatively inaccessible, and so is not at great risk of increased disturbance. Given there are a number of populations around coastal northern Palawan, and the fact the species was observed growing near a rubbish dump in El Nido town, we consider *B*. *elnidoensis* to belong the Least Concern IUCN category [48].

**Other specimens.** PHILIPPINES. Palawan, Miniloc Island, around lagoon areas on limestone, N 11°09.0', E 119°18.8', 24 Apr. 1997, Madulid, Reynoso & Agoo PPI27564 (BRIT, L); Palawan, Jul. 1912, Fenix 15540 (B, BM).

**Etymology.** Named after its type locality, El Nido Town, where the new species was discovered.

**Notes.** The new species is allied to *Begonia wadei* in the thick-trunked stem, differing in the widely to very widely ovate or subtriangular leaves, (vs. obliquely ovate), 12–30 × 10.5–25 cm (vs. 6–20 × 3–13 cm), secondary veins 3 or 4 (vs. *ca*. 6) on each side of midrib; glabrous petioles (vs. puberulous to tomentose); inflorescence 20–40 cm (vs. 6–20 cm) long, bracts to 30 × 14 mm (vs. 19 × 10 mm); capsules 10–13 × 8–13 (vs. 15–17 × 20–22 mm).

**Fig 7:**
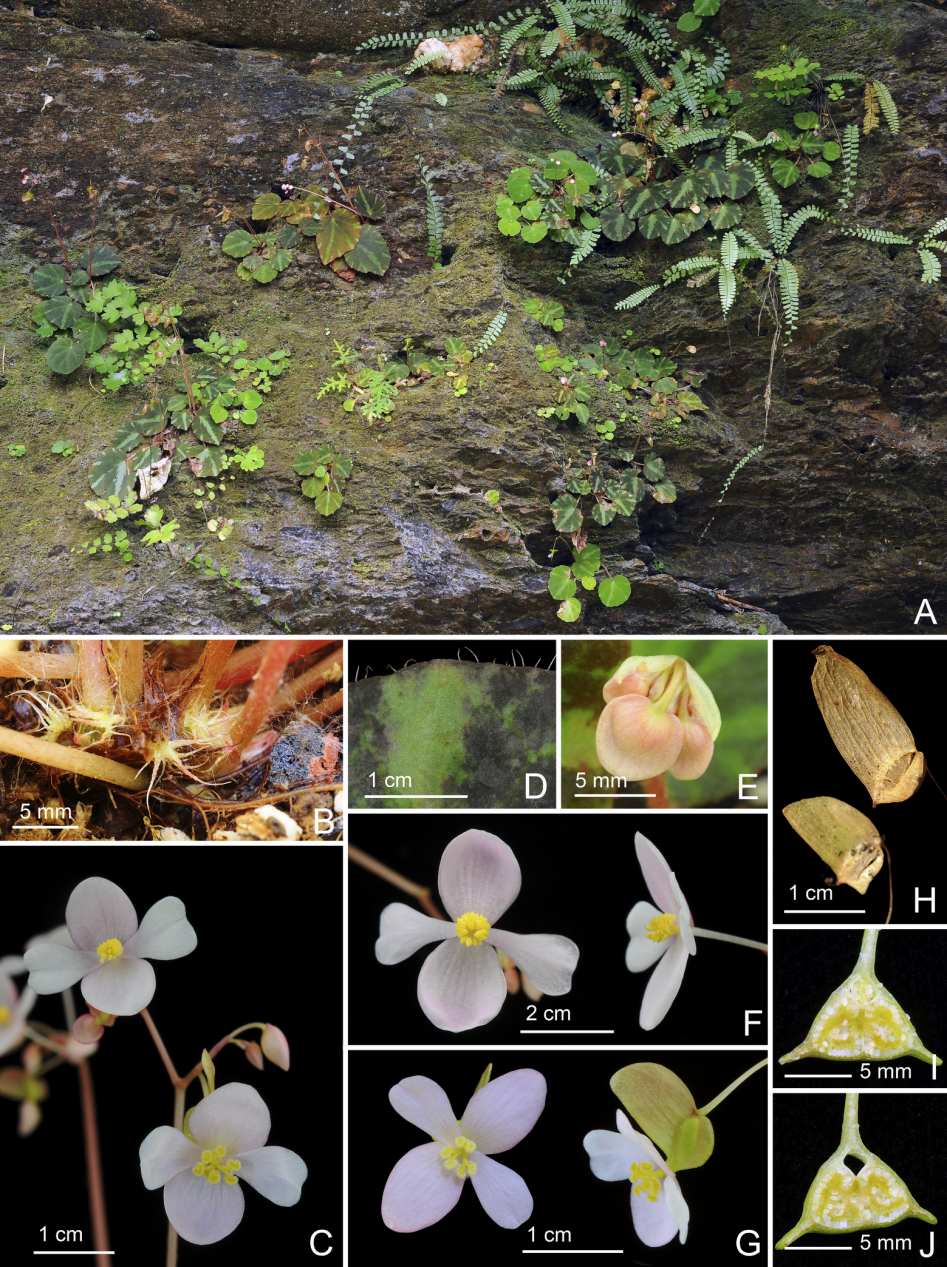
*Begonia gironellae* C.I Peng, Rubite & C.W.Lin. **A,** habitat; **B,** rhizome showing petiole base; **C,** inflorescence, showing staminate and pistillate flowers; **D,** portion of leaf, showing velutinous margin; **E,** bracts on an immature inflorescence; **F,** staminate flowers; **G,** pistillate flowers; **H,** mature capsules; **I, J,** serial cross section of an ovary. All from *Peng 24579*.

*Begonia gironellae* C.I Peng, Rubite & C.W.Lin, sp. nov. [urn:lsid:ipni.org:names:77176486–1] (Figs 2 and 7). Type:—PHILIPPINES. Palawan Island, Puerto Princesa City, Barangay Tanabag, on rocky slope along the curvy Tanabag River, exposed to semiexposed, elevation ca. 30 m, N 10°0'32", E 118°58'58", 19 Dec 2014, *C*.*-I Peng 24579* with C.-I Huang, R. R. Rubite, E. P. Gironella, M. A. Suzuki, T. Cardona (HOLOTYPE: PNH; ISOTYPE: HAST).

Monoecious stemless herb. Rhizome short, creeping, 3−5 cm, 5−8 mm thick, internodes very congested. Stipules persistent, pale yellowish-green or pinkish, widely triangular, 5–6 mm long, 6–7 mm wide, herbaceous, strongly keeled, very sparsely velutinous or nearly glabrous, margin entire, apex aristate, arista 2–3 mm long. Leaves alternate, petiole terete, pale yellowish-green to reddish, (4−)8−16 cm long, (2−)3.5−5 mm thick, sparsely woolly, glabrescent, fleshy hairs fused into a ring at the base of the petiole; leaf blade asymmetric, oblique, ovate to widely ovate, occasionally polygonal, 6−11 cm long, 4.7–9.5 cm wide, broad side 2.8–6 cm wide, base cordate, 1.2–3.5 cm long, apex obtuse to acute, margin denticulate, sparsely short-velutinous, hairs white; leaves thickly chartaceous, succulent, adaxially green, dark olive green to maroon, variegated with silver grey to pale green zones along midrib and toward the end of primary veins near leaf margin, sometimes lamina uniformly dusky green to maroon or with undertint venation; surface glabrous; abaxially pale green or reddish, sparsely velutinous on all veins; venation palmate with *ca*. 8 veins, midrib distinct, with *ca*. 2 secondary veins on each side, other primary veins branching dichotomously or nearly so, tertiary veins reticulate. Inflorescences an axillary, bisexual and protandrous, cymosely branching panicle 17–41 cm long, peduncle 14.5–31 cm long, dichasial cymes, arising directly from rhizome, branched 3–6 times, erect or ascending, yellowish-green to reddish, sparsely woolly, glabrescent. Bracts pale yellowish-green, hyaline, deciduous, those at basal node of inflorescence ovate to widely ovate, boat-shaped, 6–9 mm long, *ca*. 4 mm wide, apex obtuse or acute to mucronate, margin entire; bracts at summit of inflorescence ovate, 2–3 mm long, 1.5–2 mm wide, apex attenuate to retuse, margin entire. Staminate flower: pedicel 0.9–2.1 cm long, glabrous, tepals 4, white to pinkish, glabrous, outer 2 widely ovate or obovate to suborbicular, 0.9–1.3 cm long, 0.7–1 cm wide, inner 2 obovate or oblanceolate, 1–1.5 cm long, 0.6–1 cm wide, apex truncate to retuse; androecium sub-zygomorphic, *ca*. 0.4 cm across; stamens yellow, 32–46; filaments shortly fused at base; anthers obovate, *ca*. 0.8 mm long, apex apiculate, subequal to filaments. Pistillate flower: pedicel 1.4–3.3 cm long, glabrous; ovary pale green to reddish, body trigonous-ellipsoid, 4–6.5 mm long, 1.5–3 mm thick (wings excluded), glabrous; 3-winged, wings 6–7.5 mm long, lateral wings narrower, narrowly crescent-shaped, 1.5–3 mm wide, abaxial wing much protruded, narrowly triangular, more or less truncate distally, rounded proximally, 7–11 mm wide, margin entire; ovary 3-locular, bilamellate, abaxial locule abortive or underdeveloped; tepals 4, white to pinkish, glabrous, outer 2 obovate, widely ovate or obovate, 1–1.3 cm long, 0.8–1 cm wide, inner 2 oblanceolate to widely obovate, 1–1.5 cm long, 0.6–1 cm wide, apex truncate to retuse; styles 3, shortly fused at base, yellow, *ca*. 0.4 cm long, stigma spirally twisted. Capsule pendent, pedicel 1–1.9 cm long, tepals deciduous; body trigonous-ellipsoid, 6–9 mm long, 3–4 mm thick (wings excluded), greenish or reddish when fresh; wings unequal, lateral wings 2–4 mm wide, abaxial wing 9–16 mm wide.

**Distribution and ecology.** Endemic to Tanabag, Puerto Princesa in northern Palawan, the Philippines; occurring on riverbanks or steep mossy slopes at ca. 30 m elevation.

**Provisional conservation assessment.** Two populations were observed by the authors at the type locality at the lower reaches of the Tanabag River, one of which was locally abundant. The species is very likely to grow further up the meandering and relatively inaccessible riverbanks. If further populations exist upstream, the species could belong to the Least Concern category, or if the populations we observed are the only ones existing then VUD2 would be appropriate. Given this uncertainty we assign *B*. *gironellae* to the Data Deficient category.

**Etymology.** The species is named in honor of Prof. Elizabeth P. Gironella of the Palawan State University, who accompanied and guided us during the field trip to the type locality and southern Palawan.

**Notes.** The new species was found only in a limited area. Most plants in this population have variegated leaves with pale patches on the margin and midrib on upper surface. The widely ovate, variegated leaves resemble those of *Begonia cleopatrae*, a beautiful species also from northern Palawan. Both species also have fleshy hairs fused into a ring at the base of the leaf petiole. However, *Begonia gironellae* is distinct from *B*. *cleopatrae* in its rosette habit with rhizome shorter to 5 cm (vs. over 40 cm) long, internodes very congested (vs. *ca*. 1 cm), stipules widely triangular (vs. lanceolate), 5–6 × 6–7 mm (*ca*. 6 × 3 mm), lamina to 12.5 × 9.5 cm (vs. 6 × 6 cm), bracts 6–9 × 4–4.5 mm (vs. 3–4 × 2 mm), capsule with wider abaxial wing (to 16 mm vs. 12 mm). Additionally, *B*. *gironellae* is a lowland species occurring in broadleaved forest at seaside, whereas *B*. *cleopatrae* grows on hill forest at *ca*. 400 m altitude. An apparent difference in leaf texture was investigated using cryo-scanning electron microscopy, following the methods in Hughes & al. (2011). The lamina thickness of *B*. *cleopatrae* was ca. 700 μm, whilst that of *B*. *gironellae* was ca. 550 μm.

**Fig 8:**
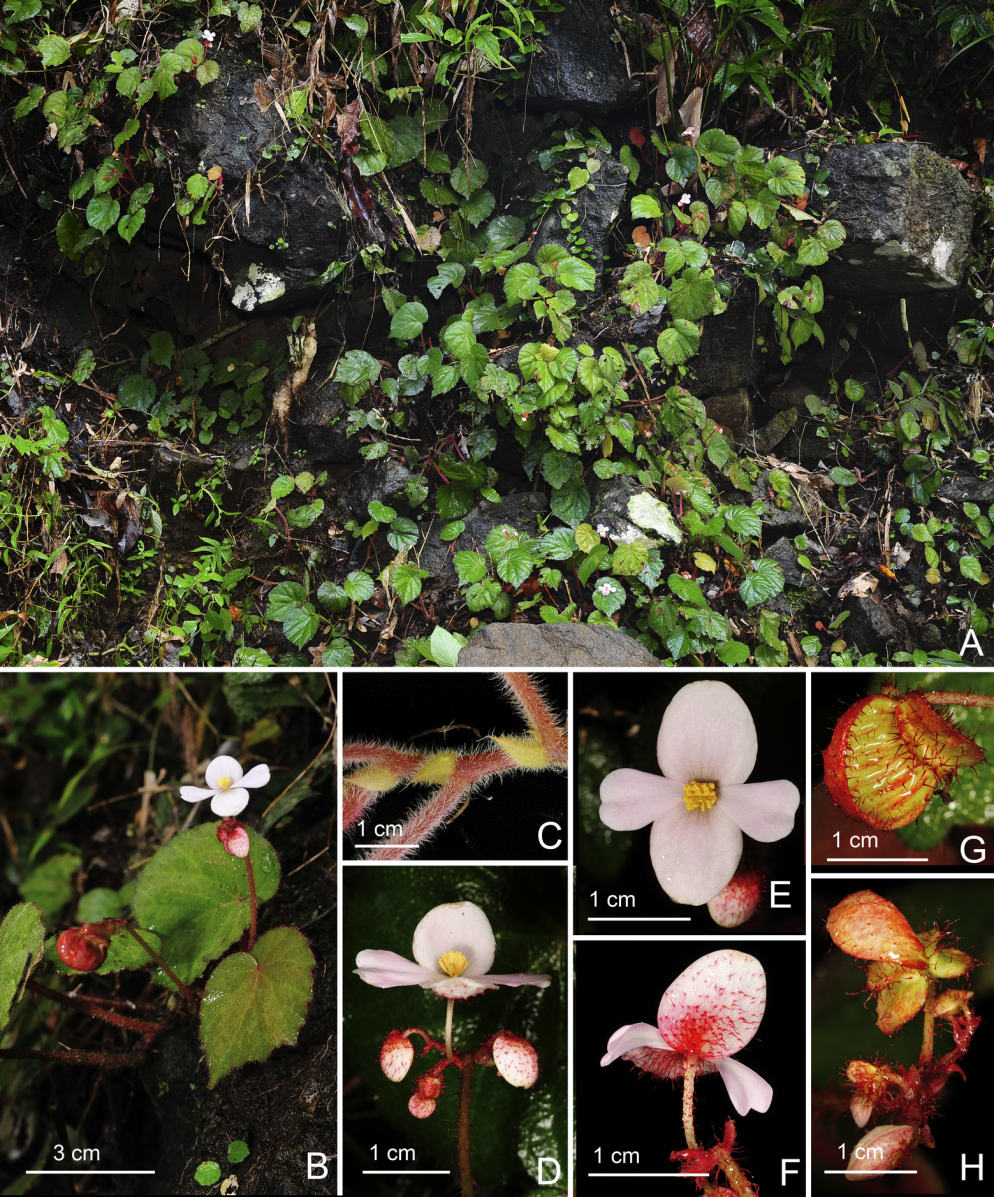
*Begonia quinquealata* C.I Peng, Rubite & C.W.Lin. **A,** habitat; **B,** habit; **C,** stipules on rhizome; **D,** inflorescence with staminate flower; **E, F,** staminate flower, face and side views; **G, H,** capsule, viewed from side and bottom. All from *Peng 24588*.

*Begonia quinquealata* C.I Peng, Rubite & C.W.Lin, sp. nov. [urn:lsid:ipni.org:names:77176487–1] (Figs 3 and 8). Type:—PHILIPPINES. Palawan Island, Puerto Princesa City, Barangay Napsan, near Salakot Falls, on grassy, rocky slope along paved road, semishaded broadleaved forest margin, elevation ca. 315 m, 9°41'58"N, 118°31'18"E, 20 Dec 2014 (fl. and fr.), *C*.*-I Peng 24588* with C.-I Huang, R. R. Rubite, E. P. Gironella, M. A. Suzuki, T. C. B. Cardona (HOLOTYPE: PNH; ISOTYPE: HAST).

Monoecious rhizomatous herb. Rhizome long creeping, to 40 cm or longer, 3−5 mm thick, densely hirsute, hairs pale magenta, internodes 1.5−5 cm long. Stipules persistent, pale green to reddish, triangular, 6–10 mm long, 4–8 mm wide, herbaceous, strongly keeled, densely hirsute, margin entire, apex aristate, arista 3–5 mm long. Leaves alternate, petiole terete, reddish, 3.2−8 cm long, 3−4 mm thick, densely hirsute; leaf blade asymmetric, oblique, widely ovate, 6.5−10.2 cm long, 4.7–7.8 cm wide, broad side 3–4.8 cm wide, basal lobes cordate, 1.7–2.8 cm long, apex acuminate to attenuate, margin denticulate and lined with white to magenta, scabrous hairs; leaf thick chartaceous, succulent, adaxially olive green; adaxially densely hirsute; abaxially pale green, hirsute on all veins; venation basally *ca*. 7 palmate, midrib distinct, *ca*. 3 secondary veins on each side, other primary veins branching dichotomously or nearly so, tertiary veins reticulate. Inflorescences an axillary, bisexual, cymosely branching panicle *ca*. 11 cm long, peduncle 8.5–10 cm long, dichasial cymes arising directly from rhizome, branched 3–4 times, erect or ascending, reddish, densely hirsute; protandrous. Bracts reddish, hyaline, deciduous, those at basal node of inflorescence ovate to lanceolate, 3–4 mm long, 1.5–2.5 mm wide, apex mucronate apiculate, margin entire or with hirsute, abaxially red hirsute or scabrous; bracts at summit of inflorescence to 1.5 mm long, 1 mm wide. Staminate flower: pedicel 0.5–0.6 cm long, scabrous, tepals 4, white to pinkish, outer 2 widely ovate or obovate to suborbicular, *ca*. 1 cm long across, abaxially red scabrous, inner 2 obovate or oblanceolate, 1–1.3 cm long, 0.5–0.6 cm wide, apex truncate to retuse; androecium sub-zygomorphic, *ca*. 0.4 cm across; stamens yellow, 25–30; filaments shortly fused at base; anthers obovate, *ca*. 0.8 mm long, 2-locular, apex apiculate, more or less equal to filaments. Pistillate flower (immature): pedicel 0.5–0.8 cm long, red scabrous; ovary yellowish-green to red, body trigonous-ellipsoid, *ca*. 7 mm long, 4 mm thick (wings excluded), sparsely red scabrous; 5-winged, wings unequal, *ca*. 1 cm long, lateral wings narrower, narrowly sub-trapezoid, *ca*. 3 mm wide, abaxial wing crescent-shaped, cucullate, *ca*. 5 mm wide, margin red scabrous, additional wing *ca*. 1 mm wide; outer tepals 2, abaxially red scabrous. Capsule pendent, pedicel 11–12 mm long, tepals deciduous; body trigonous-ellipsoid, 12–14 mm long, *ca*. 5 mm thick (wings excluded), greenish or reddish when fresh; wings 5, unequal, red scabrous, lateral wings 3–4 mm wide, abaxial wing *ca*. 9 mm wide, strongly cucullate, additional 2 side-wings 1–2 mm wide.

**Distribution and ecology.** *Begonia quinquealata* is endemic to Palawan. On low vertical cliff face or rock-strewn at base of cliffs, broadleaved forest margin.

**Provisional conservation assessment.** The species exists as a single population at the type and only locality, with ca. 100 individuals in an area several metres square. Searches around the area failed to locate further individuals. The population is near a drainage channel along a roadside, where the overhanging vegetation is being cleared. We consider *Begonia quinquealata* to belong to the Critically Endangered category, under criteria CRB2abiii&v (area of occupancy <10km2, a single location, and a continuing decline in the area and quality of habitat, and the number of mature individuals).

**Etymology.** The specific epithet refers to the 5-winged capsule.

**Notes.** *Begonia quinquealata* is very distinctive and quite unlike any other member of *Begonia* sect. *Baryandra* from Palawan in being a densely hairy herb with long creeping rhizomes. Together with *Begonia suborbiculata* Merr., they are the only two species in the Philippines with 5-winged capsules. However, *B*. *quinquealata* differs from *B*. *suborbiculata* in having widely ovate (vs. suborbicular) leaves that are thickly chartaceous (vs. fleshy), upper surface uniformly green (vs. variegated) and densely velutinous (vs. subglabrous), margin denticulate (vs. entire); outer tepals, ovary and capsules red scabrous (vs. white velutinous).

**Fig 9:**
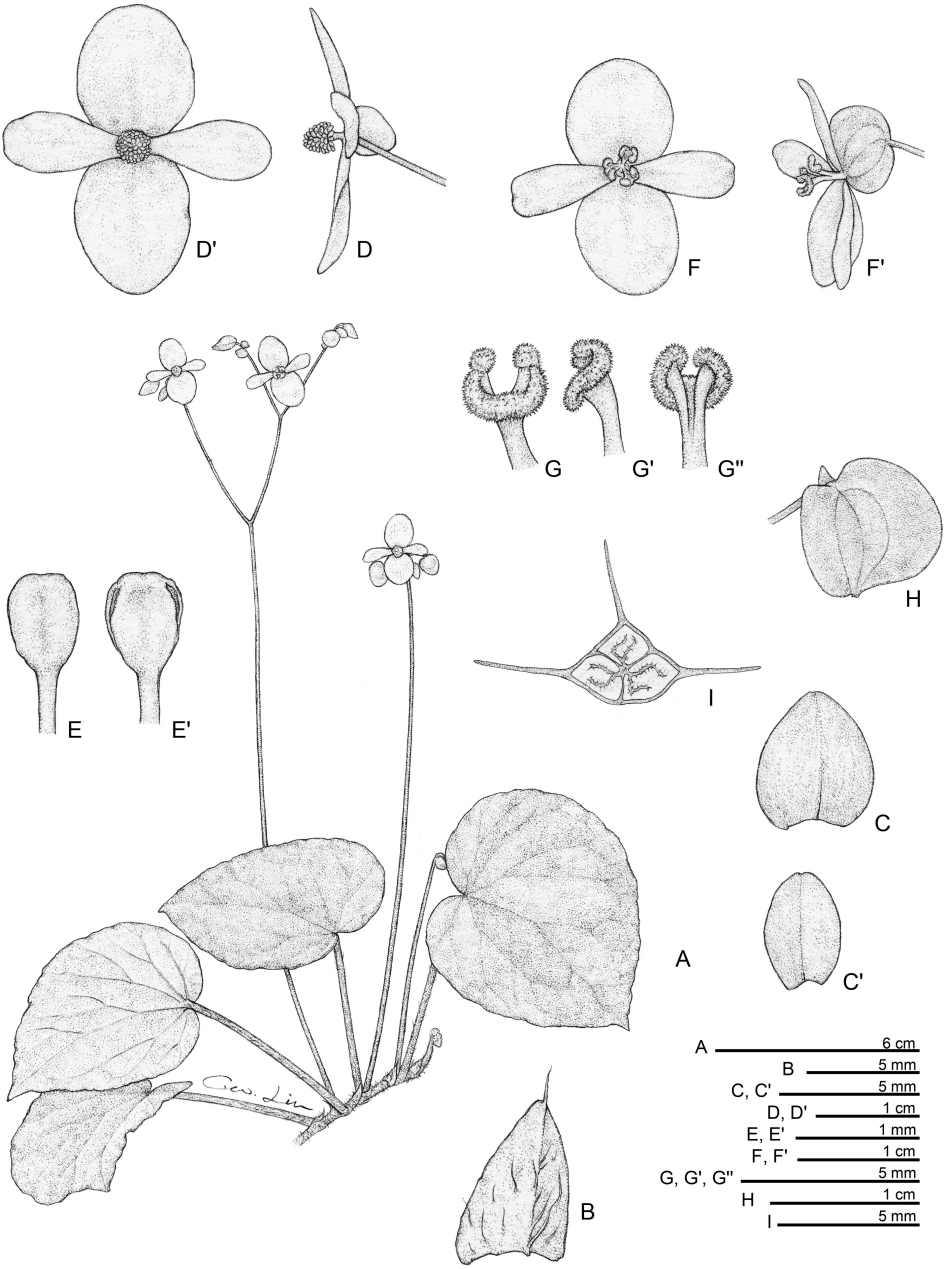
*Begonia tabonensis* C.I Peng, Rubite & C.W.Lin. **A,** habit; B, stipule; **C, C**', bracts at lowermost and upper inflorescence; **D, D',** staminate flower, face and side views; **E, E**', stamen, dorsal and ventral views; **F, F',** pistillate flower, face and side views; **G, G', G'',** style and stigmatic band, dorsal, side and ventral views; **H,** capsule; **I,** cross section of an immature capsule. All from *Peng 24538*.

**Fig 10:**
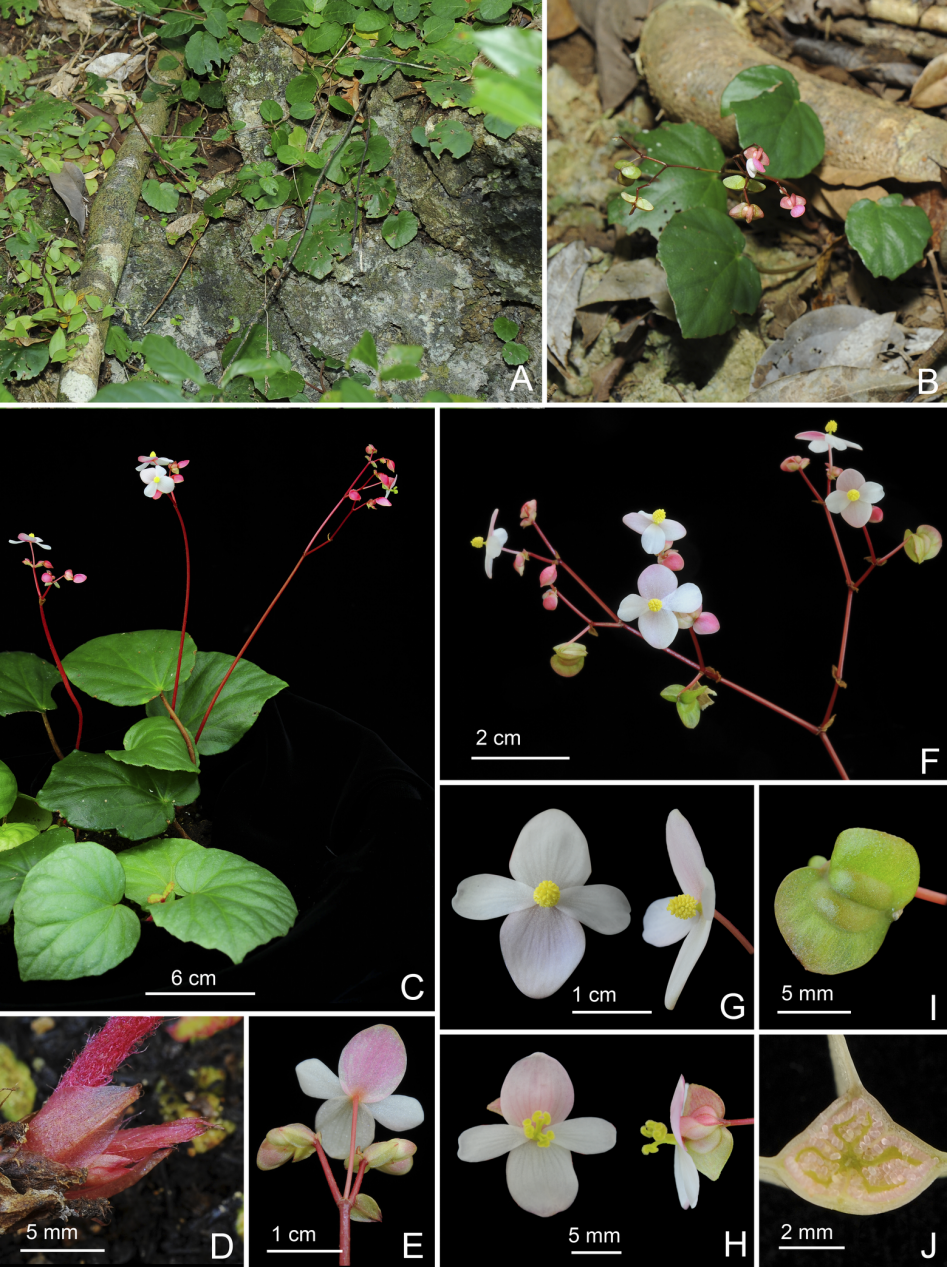
*Begonia tabonensis* C.I Peng, Rubite & C.W.Lin. **A,** Habitat; **B, C,** habit; **D,** stipules; **E,** inflorescence, showing bracts and staminate flowers; **F,** inflorescence; **G,** staminate flower, face and side views; **H,** pistillate flower, face and side views, **I,** capsule; **J,** cross section of an immature capsule. All from *Peng 24538*.

*Begonia tabonensis* C.I Peng, Rubite & C.W.Lin, sp. nov. [urn:lsid:ipni.org:names:77176488–1] (Figs 9 and 10). Type:—PHILIPPINES. Palawan Island, Municipality of Quezon, Lipuun Point, Tabon Cave, on limestone cliffs or rocky slope around the cave, elevation ca. 30 m, N 9°16'45", E 117°58'54", 13 Dec 2014, *C*.*-I Peng 24538* with C.-I Huang, R. R. Rubite, E. P. Gironella, M. A. Suzuki, T. C. B. Cardona (HOLOTYPE: PNH; ISOTYPE: HAST).

Monoecious rhizomatous herb. Rhizome creeping, 3−7 cm or longer, 3−5 mm thick, internodes to 15 mm long. Stipules persistent, reddish, triangular, 5–8 mm long, 3–5 mm wide, herbaceous, strongly keeled, abaxially sparsely velutinous, densely velutinous along midrib, margin entire, apex aristate, arista 2–3 mm long. Leaves alternate, petiole terete, reddish, 3.5−10 cm long, 2−2.5 mm thick, villous; leaf blade asymmetric, oblique, widely ovate, 4−8 cm long, 4–6.4 cm wide, broad side 2.5–3.5 cm wide, basal lobes cordate, 0.9–1.5 cm long, apex acuminate or acute, margin subentire or inconspicuously denticulate, ciliate, hairs white; leaves thickly chartaceous, adaxially grey green, glabrous; abaxially pale green, appressed velutinous on all veins; venation basally *ca*. 7 palmate, midrib distinct, *ca*. 2 secondary veins on each side, other primary veins branching dichotomously or nearly so, tertiary veins reticulate. Inflorescences a axillary, bisexual, cymosely branching panicle 9–24 cm long with dichasial cymes, peduncle 6–15 cm long, arising directly from rhizome, inflorescence branched 3–4 times, erect or ascending, crimson, glabrous; cymes glabrous or very sparsely minutely glandular; protandrous. Bracts pale yellowish-green to pinkish, hyaline, chartaceous, very sparsely glandular or glabrous, deciduous, those at basal node of inflorescence very widely ovate, *ca*. 5–6 mm across, apex mucronate apiculate or slightly retuse, margin entire; bracts at summit of inflorescence elliptic or ovate, 3–6 mm long, 1.5–3.5 mm wide, apex acute to retuse, margin entire. Staminate flower: pedicel 0.7–1.7 cm long, very sparsely glandular or glabrous, tepals 4, white to pinkish, outer 2 ovate or obovate to suborbicular, 0.7–1.1 cm long, 0.7–1 cm wide, abaxially glandular, inner 2 oblanceolate, 0.9–1 cm long, 0.4–0.6 cm wide, apex obtuse to retuse; androecium actinomorphic, *ca*. 3 mm across; stamens yellow, 60–80; filaments fused on a short stalk; anthers obovate, *ca*. 0.7 mm long, 2-locular, apex rounded to truncate, subequal to filaments. Pistillate flower: pedicel 1–1.5 cm long, glabrous or sparsely glandular; ovary pale green to pinkish, body trigonous-ellipsoid, 4–6 mm long, 3–4 mm thick (wings excluded), sparsely glandular; 3-winged, wings sub-equal, *ca*. 8 mm long, crescent-shaped, occasionally slightly pointed at summit; lateral wings 3–4 mm wide, abaxial wing 2–3 mm wide, margin entire; ovary 3-locular, bilamellate; tepals 4, white to pinkish, outer 2 obovate or suborbicular, 6.5–8 mm long, 6–7 mm wide, abaxially sparsely glandular, inner 2 oblanceolate, 6.5–8 mm long, *ca*. 3 mm wide, apex obtuse or retuse; styles 3, shortly fused at base, yellow, *ca*. 4 mm long, stigma spirally twisted. Capsule pendent, pedicel 10–16 mm long, tepals deciduous; body trigonous-ellipsoid, *ca*. 6 mm long, 5 mm thick (wings excluded), greenish or pinkish when fresh; wings subequal, *ca*. 10 mm long, 3–5 mm wide.

**Distribution and ecology.** *Begonia tabonensis* is endemic to Lipuun Point, north of Quezon municipality, in southwestern Palawan Island. It occurs on semishaded limestone cliffs around Tabon Cave, an archeological and natural heritage site, in lowland forest at *ca*. 30 m elevation.

**Provisional conservation assessment.** The species is common at the type and only locality of the Tabon Cave vicinity. The site is visited by tourists, but is sustainably managed by the local community and the National Museum Palawan Branch. Although it has a small distribution, we consider *B*. *tabonensis* to belong to the IUCN cateory Least Concern as long as the site remains undisturbed.

**Etymology.** The specific epithet refers to Tabon Cave where the new species was discovered.

**Notes.** *Begonia tabonensis* resembles *B*. *mindorensis* Merr. in the widely ovate, uniformly green leaves and inflorescence with sessile glands, differing in having shorter petioles (to 10 cm vs. 25 cm long); leaves 4−8 × 4–6.4 cm (vs. 10−15 × 6–10 cm); bracts deciduous (vs. persistent), chartaceous (vs. coriaceous), glabrous or very sparsely glandular (vs. densely glandular); ovary wing crescent-shaped, sometimes slightly pointed at summit (vs. triangular, acute at summit).

**Fig 11:**
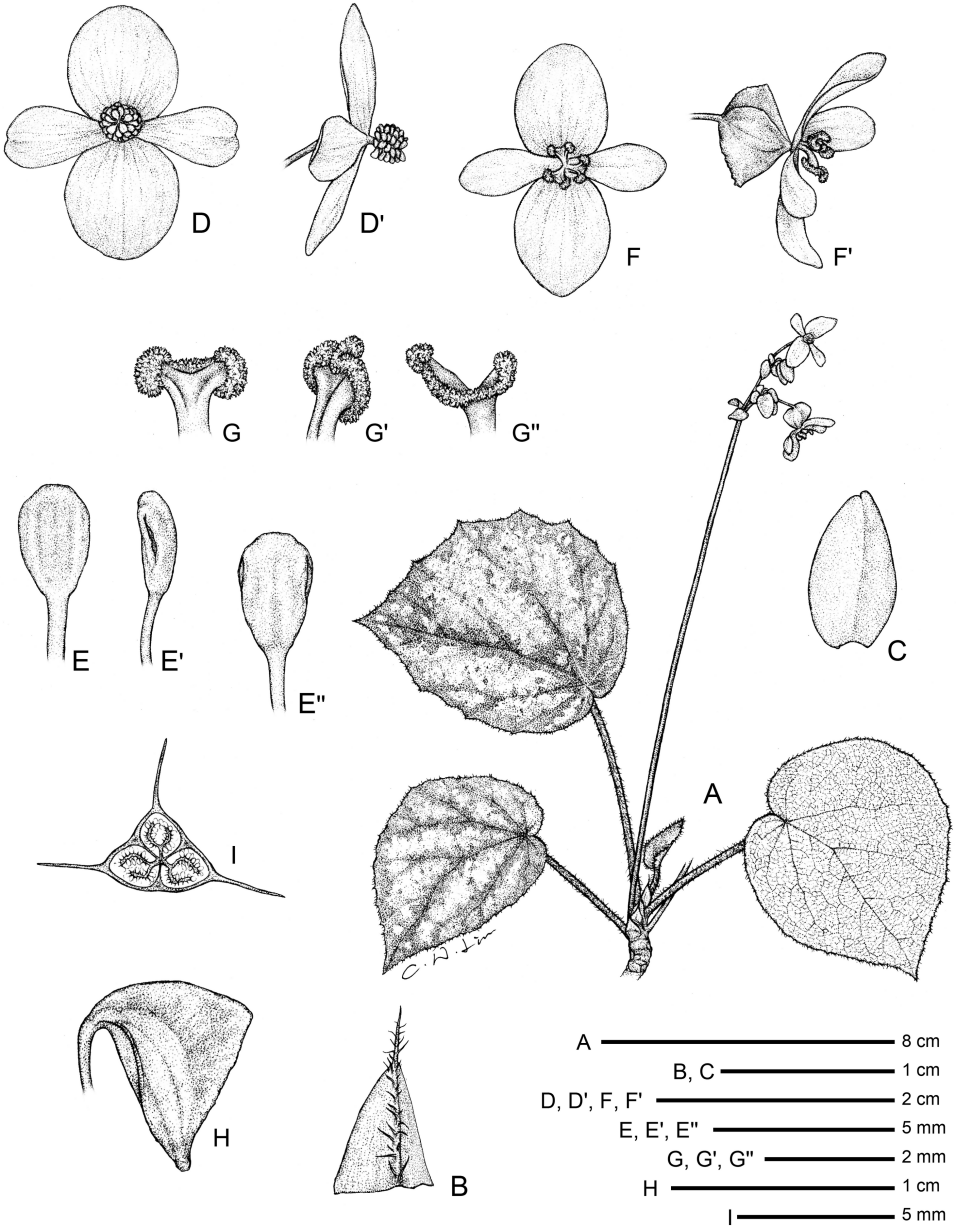
*Begonia tenuibracteata* C.I Peng, Rubite & C.W.Lin. **A,** habit; **B,** stipule; **C,** bracts; **D, D',** staminate flower, face and side views; **E, E', E'',** stamen, ventral, side and dorsal views; **F, F',** pistillate flower, face and side views; **G, G', G'',** style and stigmatic band, ventral, side and dorsal views; **H,** capsule; **I,** cross section of an immature capsule. All from *Peng 23452*.

**Fig 12:**
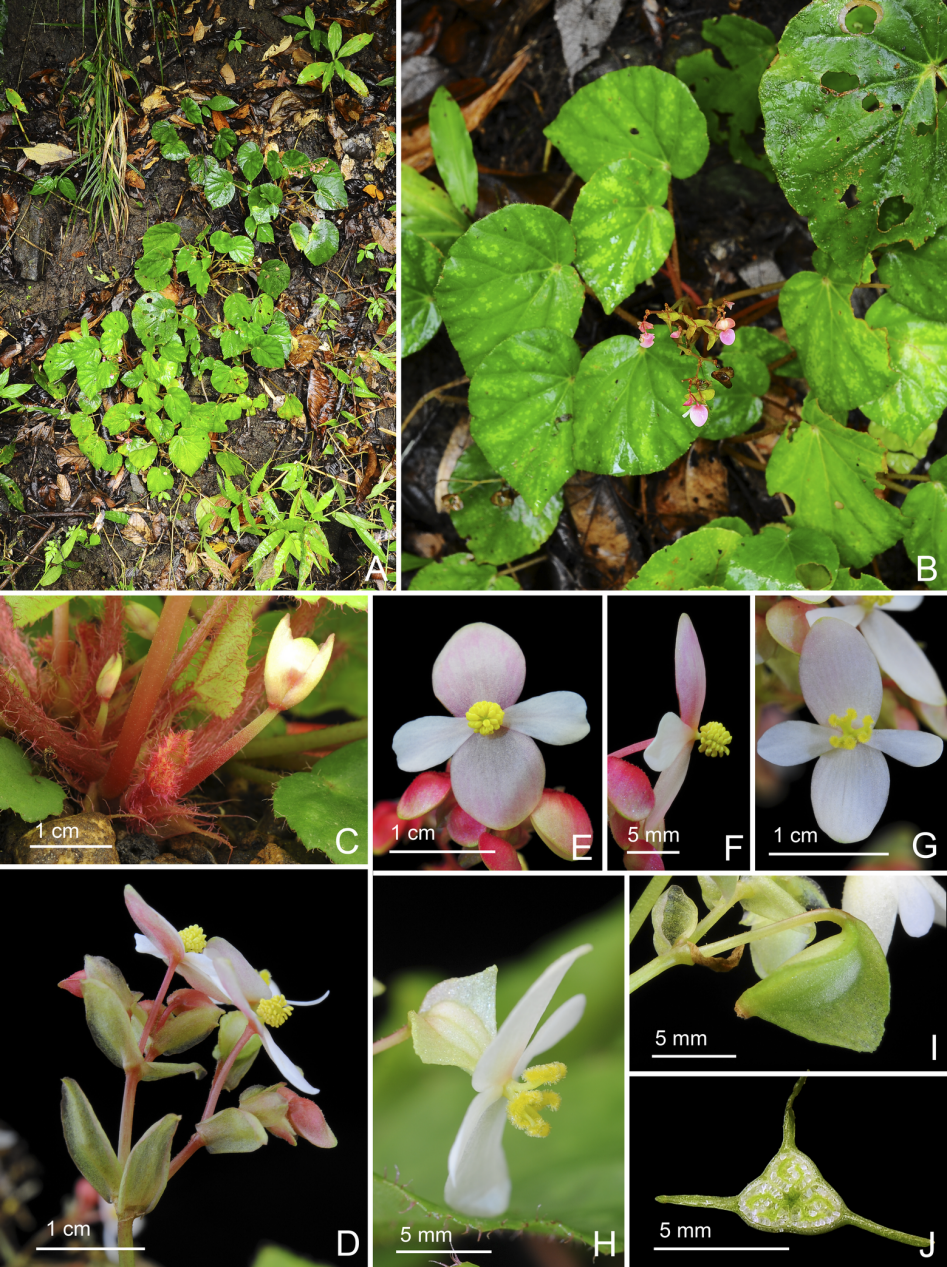
*Begonia tenuibracteata* C.I Peng, Rubite & C.W.Lin. **A,** habitat; **B,** habit; **C,** rhizome, showing stipules, petioles and immature inflorescences; **D,** inflorescence, showing hyaline bracts and staminate flowers; **E, F,** staminate flower, face and side views; **G, H,** pistillate flower, face and side views; **I,** capsule; **J,** cross section of an immature capsule. All from *Peng 23452*.

*Begonia tenuibracteata* C.I Peng, Rubite & C.W.Lin, sp. nov. [urn:lsid:ipni.org:names:77176489–1] (Figs 11 and 12). Type:—PHILIPPINES. Palawan Island, Puerto Princesa City, Barangay Napsan, near Salakot Falls, on grassy, rocky slope, exposed to semi exposed, elevation ca. 180 m, N 9°41'13", E 118°32'6", 1 Nov 2011, *C*.*-I Peng 23452* with K.-F. Chung, C.-I Huang & R. R. Rubite (HOLOTYPE: PNH; ISOTYPE: HAST).

Monoecious rhizomatous herb. Rhizome creeping, 3−10 cm or longer, 3−7 mm thick, internodes very congested, to 3 mm long. Stipules persistent, pale yellowish-green or pinkish, triangular, 5–6 mm long, 2.5–3 mm wide, herbaceous, strongly keeled, very sparsely velutinous along midrib, margin entire, apex aristate, arista 3–4 mm long. Leaves alternate, petiole terete, pale yellowish-green to reddish, 4−7 cm long, 2−2.5 mm thick, villous; leaf blade asymmetric, oblique, ovate to widely ovate, 4−8.6 cm long, 3.5–7 cm wide, broad side 2–4 cm wide, basal lobes cordate, 0.5–2 cm long, apex acuminate or acute, margin denticulate and lined with white to magenta, sparsely velutinous hairs; leaf thickly chartaceous, succulent, adaxially green, sometimes with irregular pale green to silvery variegations between veins, surface glabrous or very sparsely velutinous; abaxially pale green,velutinous on all veins; venation basal, *ca*. 7 palmate, midrib distinct, *ca*. 2 secondary veins on each side, other primary veins branching dichotomously or nearly so, tertiary veins reticulate. Inflorescences an axillary, bisexual, cymosely branching panicle 7–22 cm long with dichasial cymes, peduncle 5–17 cm long, arising directly from rhizome, branched 3–4 times, erect or ascending, yellowish-green to reddish, glabrous or very minutely puberulous; cymes glabrous or very sparsely sessile-glandular; protandrous. Bracts pale yellowish-green, hyaline, nearly membranaceous, very sparsely glandular or glabrous, usually persistent, those at basal node of inflorescence widely lanceolate, 6–12 mm long, 3–6 mm wide, apex mucronate apiculate or slightly retuse, margin entire; bracts at summit of inflorescence 2.5–4 mm long, 1.7–2.5 mm wide, apex acute to retuse, margin entire. Staminate flower: pedicel 0.8–2 cm long, glabrous or with sparse, sessile glands, tepals 4, white to pinkish, outer 2 ovate or obovate to suborbicular, 0.8–1 cm long, 0.5–8 cm wide, abaxially glandular, inner 2 oblanceolate, 0.6–0.9 cm long, 0.2–0.4 cm wide, apex obtuse to rounded; androecium actinomorphic, *ca*. 0.3 cm across; stamens yellow, 40–48; filaments shortly fused at base; anthers obovate, *ca*. 0.6 mm long, 2-locular, apex rounded to truncate, subequal to filaments. Pistillate flower: pedicel 0.8–1 cm long, glabrous or with sparse, sessile glands; ovary pale green to pinkish, body trigonous-ellipsoid, 4–6 mm long, 2–3.5 mm thick (wings excluded), sparsely glandular; 3-winged, wings subequal, 6–7 mm long, lateral wings narrower, shallowly sub-triangular, 1–4 mm wide, abaxial wing shallowly sub-triangular, rounded proximally, 1.5–4.5 mm wide, margin entire; ovary 3-locular, placentation bilamellate; tepals 4, white to pinkish, outer 2 ovate or obovate, 6.5–8 mm long, 5–7 mm wide, with sessile glands abaxially, inner 2 oblanceolate, 5–8 mm long, 2.5–3.5 mm wide, apex obtuse; styles 3, shortly fused at base, greenish-yellow, *ca*. 3 mm long, stigma spirally twisted. Capsule pendent, pedicel 7–10 mm long, tepals deciduous; body trigonous-ellipsoid, ca. 7 mm long, 3 mm thick (wings excluded), greenish or pinkish when fresh; wings subequal, *ca*. 9 mm long, lateral wings 3–4 mm wide, abaxial wing 3.5–4 mm wide.

**Distribution and ecology.** *Begonia tenuibracteata* is endemic to central Palawan, occurring on mossy boulders along road cut in shaded, wet lowland forest, near Salakot Falls, Napsan.

**Provisional conservation assessment.** The species is locally abundant at the type and only locality, however the number of individuals in the Salakot Falls area has decreased significantly according to our observations at the site in 2006 and 2011. We consider *B*. *tenuibracteata* to belong to the IUCN category Endangered (ENC1) due to this observed decline.

**Etymology.** The specific epithet refers to the hyaline, membranaceous bracts of this new species.

**Notes.** *Begonia mindorensis*, a widespread species in lowland to montane forests in Luzon, Mindoro and Palawan, also produces the unusual, conspicuous, persistent bracts on the inflorescences like *B*. *tenuibracteata*. However, *B*. *tenuibracteata* is sharply distinct by the ovate to lanceolate (vs. widely to depressed ovate) bracts that are hyaline, membranaceous (vs. coriaceous), glabrous or with very sparse sessile glands (vs. densely clothed with sessile glands). In addition, *B*. *tenuibracteata* differs by the congested rhizomes with internodes only to 3 mm (vs. 20 mm) long; shorter petioles (to 7 cm vs. 10–25 cm long); velvety (vs. glossy) leaf upper surface; shorter inflorescence (to 22 cm vs. over 35 cm long); and fewer stamens (40–50 vs. *ca*. 70).

## Supporting information

S1 Table Genbank accession numbers and voucher information for the DNA sequences used in the phylogenetic analyses. Samples newly sequenced for this study are highlighted with an asterisk.
(PDF)

S1 Fig A majority rule consensus tree based on both nuclear and plastid phylogenies. The consensus combines all the post burn-in trees resulting from both the Bayesian phylogenetic analyses (chloroplast and ITS data). The nodes are labelled with internode certainty (IC) values.
(TIF)
