# Supplementary material for: Chloroplast and nuclear DNA exchanges among Begonia sect. Baryandra species (Begoniaceae) from Palawan Island, Philippines, and descriptions of five new species
Source: PLoS One. 2018 May 2;13(5):e0194877. doi: 10.1371/journal.pone.0194877 (PMC5931476; doi:10.1371/journal.pone.0194877)
Supplement: S1 Table — Samples newly sequenced for this study are highlighted with an asterisk. (PDF) [file pone.0194877.s001.pdf]

Tip label	Taxon	*ndhA* intron	*ndhF–rpl32*	*rpl32–trnL*	*trnC–ycf6*	*ycf6–psbM*	*psbM–trnD*	ITS	Voucher
B. acclivis1	*B. acclivis* Coyle	---	---	---	---	---	---	---	Peng 24565 (HAST)
B. acclivis2	*B. acclivis* Coyle	---	---	---	---	---	---	---	Peng 24575 (HAST)
B. acuminatissima	*B. acuminatissima* Merr.	KR186445	KR186532	KR186706	KR186792	KR186879	KR186619	KR186965	Rubite R321 (PNH)
B. aff. gueritziana	*B.* aff. *gueritziana* Gibbs	KR186446	KR186533	KR186707	KR186793	KR186880	KR186620	KR186966	Peng P22344 (HAST)
B. albococcinea	*B. albococcinea* Hook.	KR186447	KR186534	KR186708	KR186794	KR186881	KR186621	no data	Peng P23302 (HAST)
B. anisoptera	*B. anisoptera* Merr.	KR186448	KR186535	KR186709	KR186795	KR186882	KR186622	JX656720	Rubite R479 (PNH)
B. biliranensis	*B. biliranensis* Merr.	KR186449	KR186536	KR186710	KR186796	KR186883	KR186623	KR186967	Rubite R311 (PNH)
B. blancii	*B. blancii* M. Hughes & C.-I Peng	KR186450	KR186537	KR186711	KR186797	KR186884	KR186624	KR186968	Peng P22545 (HAST)
B. bonthainensis	*B. bonthainensis* Hemsl.	KR186451	KR186538	KR186712	KR186798	KR186885	KR186625	no data	Peng P22531 (HAST)
B. calcicola	*B. calcicola* Merr.	KR186452	KR186539	KR186713	KR186799	KR186886	KR186626	JX656708	Peng P20761 (HAST)
B. camiguinensis	*B. camiguinensis* Elmer	KR186453	KR186540	KR186714	KR186800	KR186887	KR186627	JX656721	Rubite R506 (PNH)
B. cf. nigritarum	*B.* cf. *nigritarum* (Kamel) Steud.	---	---	---	---	---	---	---	Peng 24591 (HAST)
B. chingipengii	*B. chingipengii* Rubite	KR186455	KR186542	KR186716	KR186802	KR186889	KR186629	KR186970	Peng P23368 (HAST)
B. chloroneura	*B. chloroneura* P.Wilkie & Sands	KR186456	KR186543	KR186717	KR186803	KR186890	KR186630	KR186971	Wilkie et al., 29015 (E)
B. cleopatrae	*B. cleopatrae* Coyle	KR186457	KR186544	KR186718	KR186804	KR186891	KR186631	KR186972	Wilkie et al., 25373 (E)
B. culasiensis1	*B. culasiensis* ined.	KR186458	KR186545	no data	KR186805	KR186892	KR186632	KR186973	Peng P23793 (HAST)
B. culasiensis2	*B. culasiensis* ined.	KR186459	KR186546	KR186719	KR186806	KR186893	KR186633	KR186974	Rubite R234 (PNH)
B. dipetala	*B. dipetala* Graham	KR186460	KR186547	KR186720	KR186807	KR186894	KR186634	no data	Peng P22520 (HAST)
B. dregei	*B. dregei* Otto & A.Dietr.	KR186461	KR186548	KR186721	KR186808	KR186895	KR186635	no data	Peng P20868 (HAST)
B. elmeri	*B. elmeri* Merr.	KR186462	KR186549	KR186722	KR186809	KR186896	KR186636	JX656714	Rubite R319 (PNH)
B. elnidoensis	*B. elnidoensis* C.-I Peng, R. Rubite & C. W. Lin	KR186463	KR186550	KR186723	KR186810	KR186897	KR186637	KR186976	Peng P23508 (HAST)
B. fenecis1	*B. fenicis* Merr.	KR186464	KR186551	KR186724	KR186811	KR186898	KR186638	KR186977	Peng P10794 (HAST)
B. fenecis2	*B. fenicis* Merr.	KR186465	KR186552	KR186725	KR186812	KR186899	KR186639	KR186978	Unkn. NK11979 (HAST)
B. floccifera	*B. floccifera* Bedd.	KR186466	KR186553	KR186726	KR186813	KR186900	KR186640	no data	Peng P21216 (HAST)
B. forbesii	*B. forbesii* King	KR186467	KR186554	KR186727	KR186814	KR186901	KR186641	JX656704	Peng P22685 (HAST)
B. foxworthyii	*B. foxworthyii* Burkill ex Ridl.	KR186468	KR186555	KR186728	KR186815	KR186902	KR186642	JX656702	Peng P22721 (HAST)
B. gabaldonensis	*B. gabaldonensis* ined.	KR186469	KR186556	KR186729	KR186816	KR186903	KR186643	no data	Peng P23356 (HAST)
B. gironellae1	*B. gironellae* C.-I Peng, R. Rubite & C. W. Lin	---	---	---	---	---	---	---	Peng 24579 (HAST)
B. gironellae2	*B. gironellae* C.-I Peng, R. Rubite & C. W. Lin	---	---	---	---	---	---	---	Peng 24581 (HAST)
B. gironellae3	*B. gironellae* C.-I Peng, R. Rubite & C. W. Lin	---	---	---	---	---	---	---	Peng 24580 (HAST)
B. gueritziana1	*B. gueritziana* Gibbs	KR186471	KR186558	KR186731	KR186818	KR186905	KR186645	KR186980	Peng P22311 (HAST)
B. gueritziana2	*B. gueritziana* Gibbs	KR186472	KR186559	KR186732	KR186819	KR186906	KR186646	KR186981	Peng P22342 (HAST)
B. gutierrezii1	*B. gutierrezii* Coyle	KR186473	KR186560	KR186733	KR186820	KR186907	KR186647	KR186982	Blanc s.n. (E)
B. gutierrezii2	*B. gutierrezii* Coyle	---	---	---	---	---	---	---	Peng 24534 (HAST)
B. hernandioides1	*B. hernandioides* Merr.	KR186474	KR186561	KR186734	KR186821	KR186908	KR186648	KR186983	Peng P21006 (HAST)
B. hernandioides2	*B. hernandioides* Merr.	KR186475	KR186562	KR186735	KR186822	KR186909	KR186649	no data	Rubite R106 (PNH)
B. hughesii1	*B. hughesii* R. Rubite & C.-I Peng	KR186476	KR186563	KR186736	KR186823	KR186910	KR186650	KR186984	Peng P23466 (HAST)
B. hughesii2	*B. hughesii* R. Rubite & C.-I Peng	KR186477	KR186564	KR186737	KR186824	KR186911	KR186651	KR186985	Peng P23475 (HAST)
B. ignorata	*B. ignorata* Irmsch.	KR186478	KR186565	KR186738	KR186825	KR186912	KR186652	no data	Peng P22725 (HAST)
B. kingiana	*B. kingiana* Irmsch.	KR186479	KR186566	KR186739	KR186826	KR186913	KR186653	no data	Peng P21226 (HAST)
B. klemmei	*B. klemmei* Merr.	KR186480	KR186567	KR186740	KR186827	KR186914	KR186654	JX656709	Rubite R182 (PNH)
B. laruei	*B. laruei* M.Hughes	KR186481	KR186568	KR186741	KR186828	KR186915	KR186655	no data	Hughes MH1398 (E)
B. longiscapa1	*B. longiscapa* Warb.	KR186482	KR186569	KR186742	KR186829	KR186916	KR186656	KR186986	Rubite R298 (PNH)
B. longiscapa2	*B. longiscapa* Warb.	KR186483	KR186570	KR186743	KR186830	KR186917	KR186657	KR186987	Rubite R309 (PNH)
B. luzonensis	*B. luzonensis* Warb.	KR186484	KR186571	KR186744	KR186831	KR186918	KR186658	KR186988	Rubite R316 (PNH)
B. luzonensis2	*B. luzonensis* Warb.	KR186485	KR186572	KR186745	KR186832	KR186919	KR186659	KR186989	Rubite 420 (PNH)
B. manillensis	*B. manillensis* A.DC.	KR186486	KR186573	KR186746	KR186833	KR186920	KR186660	KR186990	Rubite R256 (PNH)
B. masoniana	*B. masoniana* Irmsch.	KR186487	KR186574	KR186747	KR186834	KR186921	KR186661	no data	Peng P21411 (HAST)
B. merrilliana	*B. merrilliana* ined.	KR186488	KR186575	KR186748	KR186835	KR186922	KR186662	KR186991	Peng P23765 (HAST)
B. mindorensis1	*B. mindorensis* Merr.	KR186489	KR186576	KR186749	KR186836	KR186923	KR186663	KR186992	Rubite R326 (PNH)
B. mindorensis2	*B. mindorensis* Merr.	KR186490	KR186577	KR186750	KR186837	KR186924	KR186664	KR187002	Peng P23456 (HAST)
B. mindorensis3	*B. mindorensis* Merr.	---	---	---	---	---	---	---	Peng 24572 (HAST)
B. mindorensis4	*B. mindorensis* Merr.	---	---	---	---	---	---	---	Peng 24586 (HAST)
B. nelumbifolia	*B. nelumbifolia* Schltdl. & Cham.	KR186491	KR186578	KR186751	KR186838	KR186925	KR186665	no data	Peng P20879 (HAST)
B. nigritarum1	*B. nigritarum* (Kamel) Steud.	KR186492	KR186579	KR186752	KR186839	KR186926	KR186666	JX656719	Rubite R419 (PNH)
B. nigritarum2	*B. nigritarum* (Kamel) Steud.	KR186494	KR186581	KR186754	KR186841	KR186928	KR186668	KR186996	Peng P23855 (HAST)
B. nigritarum3	*B. nigritarum* (Kamel) Steud.	KR186497	KR186584	KR186757	KR186844	KR186931	KR186671	KR186999	Peng P23372 (HAST)
B. nigritarum4	*B. nigritarum* (Kamel) Steud.	KR186496	KR186583	KR186756	KR186843	KR186930	KR186670	KR186998	Peng P23451 (HAST)
B. nigritarum5	*B. nigritarum* (Kamel) Steud.	KR186495	KR186582	KR186755	KR186842	KR186929	KR186669	no data	Peng P23373 (HAST)
B. nigritarum6	*B. nigritarum* (Kamel) Steud.	KR186498	KR186585	KR186758	KR186845	KR186932	KR186672	KR187000	Peng P23586 (HAST)
B. nigritarum7	*B. nigritarum* (Kamel) Steud.	KR186499	KR186586	KR186759	KR186846	KR186933	KR186673	KR187001	Peng P23858 (HAST)
B. ningmingensis	*B. ningmingensis* D. Fang, Y. G. Wei & C.-I Peng	KR186500	KR186587	KR186760	KR186847	KR186934	KR186674	no data	Peng P20322 (HAST)
B. obtusifolia	*B. obtusifolia* Merr.	KR186501	KR186588	KR186761	KR186848	KR186935	KR186675	KR186993	Peng P23828 (HAST)
B. oxysperma1	*B. oxysperma* A.DC.	KR186502	KR186589	KR186762	KR186849	KR186936	KR186676	JX656710	Rubite R213 (PNH)
B. oxysperma2	*B. oxysperma* A.DC.	KR186503	KR186590	KR186763	KR186850	KR186937	KR186677	KR186994	Peng P23015 (HAST)
B. palawanensis	*B. palawanensis* Merr.	KR186504	KR186591	KR186764	KR186851	KR186938	KR186678	no data	Peng P23453 (HAST)
B. quinquealata	*B. quinquealata* C.-I Peng, R. Rubite & C. W. Lin	---	---	---	---	---	---	---	Peng 24588 (HAST)
B. rubitae	*B. rubiteae* M.Hughes	KR186505	KR186592	KR186765	KR186852	KR186939	KR186679	KF636465	Rubite R356 (PNH)
B. rufipila	*B. rufipila* Merr.	KR186506	KR186593	KR186766	KR186853	KR186940	KR186680	JX656712	Rubite R265 (PNH)
B. sp2	*B.* sp. sect. *Baryandra*	KR186454	KR186541	KR186715	KR186801	KR186888	KR186628	no data	Rubite R98 (PNH)
B. sp3	*B.* sp. sect. *Baryandra*	KR186510	KR186597	KR186770	KR186857	KR186944	KR186684	KR187004	Rubite R136 (PNH)
B. sp4	*B.* sp. sect. *Baryandra*	KR186511	KR186598	KR186771	KR186858	KR186945	KR186685	KR187005	Rubite R290 (PNH)
B. sp6	*B.* sp. sect. *Baryandra*	KR186513	KR186600	KR186773	KR186860	KR186947	KR186687	no data	Peng P23418 (HAST)
B. sp8(1)	*B.* sp. sect. *Baryandra*	---	---	---	---	---	---	---	Peng 24577 (HAST)
B. sp8(2)	*B.* sp. sect. *Baryandra*	---	---	---	---	---	---	---	Peng 24578 (HAST)
B. sublobata	*B. sublobata* Jack	KR186515	KR186602	KR186775	KR186862	KR186949	KR186689	no data	Girm. DEDEN1486 (E)
B. subnummularifolia	*B. subnummarifolia* Merr.	KR186516	KR186603	KR186776	KR186863	KR186950	KR186690	JX656722	no voucher
B. suborbiculata	*B. suborbiculata* Merr.	KR186517	KR186604	KR186777	KR186864	KR186951	KR186691	JX656716	Rubite R353 (PNH)
B. sutherlandii	*B. sutherlandii* Hook.f.	KR186518	KR186605	KR186778	KR186865	KR186952	KR186692	no data	Jasper 1200-5 (HAST)
B. sykakeingii1	*B. sykakiengii* ined.	KR186519	KR186606	KR186779	KR186866	KR186953	KR186693	KR187006	Peng P23856 (HAST)
B. sykakeingii2	*B. sykakiengii* ined.	KR186520	KR186607	KR186780	KR186867	KR186954	KR186694	no data	Peng P23890 (HAST)
B. tabonensis	*B. tabonensis* C.-I Peng, R. Rubite & C. W. Lin	---	---	---	---	---	---	---	Peng 24538)HAST)
B. tagbanua1	*B. tagbanua* M.Hughes, C.-I Peng & R. Rubite	KR186521	KR186608	KR186781	KR186868	KR186955	KR186695	KR187008	Blanc s.n. (E)
B. tagbanua2	*B. tagbanua* M.Hughes, C.-I Peng & R. Rubite	KR186522	KR186609	KR186782	KR186869	KR186956	KR186696	KR187008	Peng P23472 (HAST)
B. taraw1	*B. taraw* M.Hughes, C.-I Peng & R. Rubite	KR186523	KR186610	KR186783	KR186870	KR186957	KR186697	KR187009	Blanc s.n. taraw2 (E)
B. taraw2	*B. taraw* M.Hughes, C.-I Peng & R. Rubite	KR186524	KR186611	KR186784	KR186871	KR186958	KR186698	no data	Blanc s.n. taraw1 (E)
B. tayabensis	*B. tayabensis* Merr.	KR186525	KR186612	KR186785	KR186872	KR186959	KR186699	JX656718	Rubite R360 (PNH)
B. tenuibracteata	*B. tenuibracteata* C.-I Peng, R. Rubite & C. W. Lin	KR186512	KR186599	KR186772	KR186859	KR186946	KR186686	no data	Peng P23452 (HAST)
B. tigrina	*B. tigrina* Kiew	KR186526	KR186613	KR186786	KR186873	KR186960	KR186700	JX656703	Peng P22720 (HAST)
B. trichocheila	*B. trichocheila* Warb.	KR186527	KR186614	KR186787	KR186874	KR186961	KR186701	KR187010	Peng P20764 (HAST)
B. wadei	*B. wadei* Merr. & Quisumb.	KR186528	KR186615	KR186788	KR186875	KR186962	KR186702	KR187011	Rubite R699 (PNH)
B. woodii1	*B. woodii* Merr.	KR186529	KR186616	KR186789	KR186876	KR186963	KR186703	KR187012	Peng P23479 (HAST)
B. woodii2	*B. woodii* Merr.	KR186530	KR186617	KR186790	KR186877	KR186964	KR186704	KR187013	Peng P23496 (HAST)
