# Supplementary figures and images for: Chloroplast and nuclear DNA exchanges among Begonia sect. Baryandra species (Begoniaceae) from Palawan Island, Philippines, and descriptions of five new species
Source: PLoS One. 2018 May 2;13(5):e0194877. doi: 10.1371/journal.pone.0194877 (PMC5931476; doi:10.1371/journal.pone.0194877)

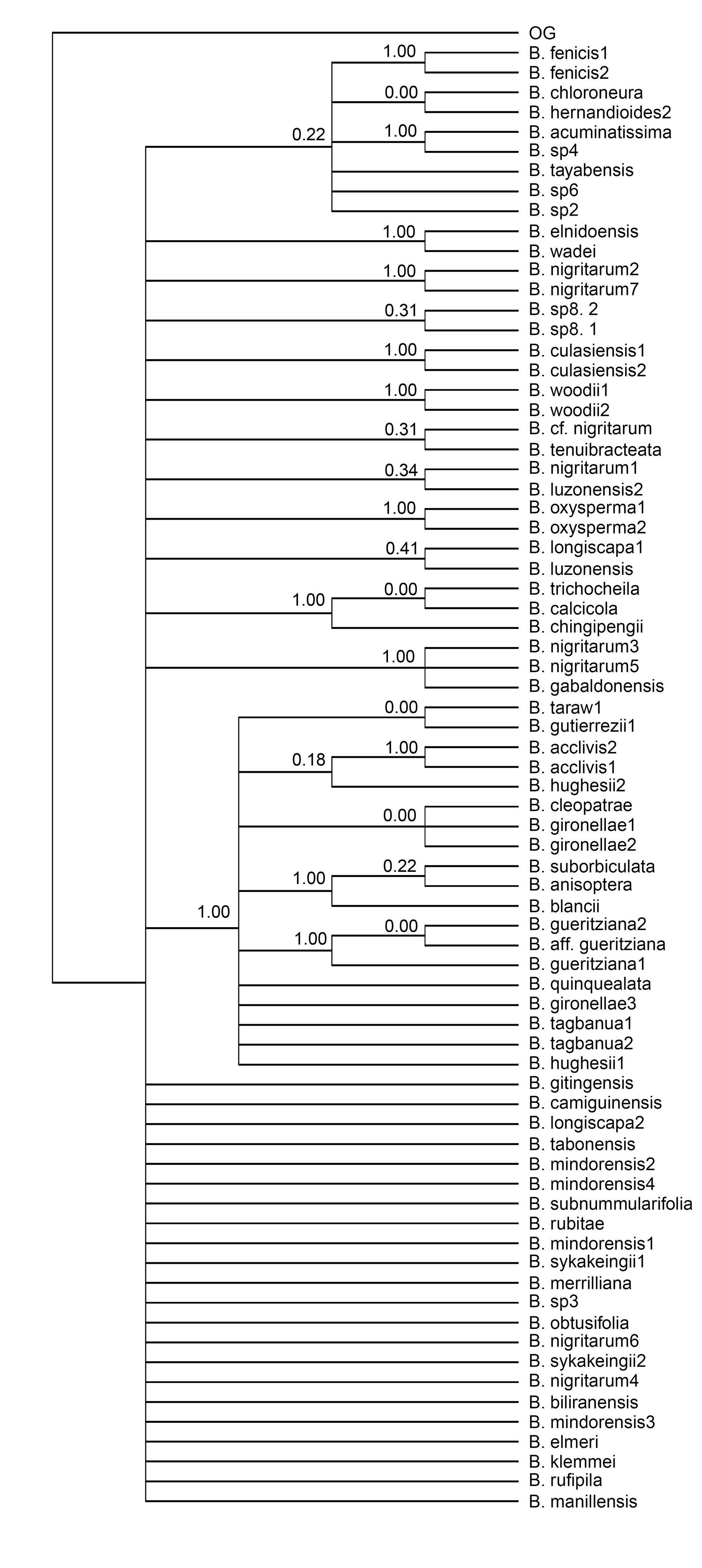

Supplement: S1 Fig — The consensus combines all the post burn-in trees resulting from both the Bayesian phylogenetic analyses (chloroplast and ITS data). The nodes are labelled with internode certainty (IC) values. (TIF) [file pone.0194877.s002.tif]
